# EZH2 mediated metabolic rewiring promotes tumor growth independently of histone methyltransferase activity in ovarian cancer

**DOI:** 10.1186/s12943-023-01786-y

**Published:** 2023-05-20

**Authors:** Jianfeng Chen, Jing Han Hong, Yulin Huang, Shini Liu, Jiaxin Yin, Peng Deng, Yichen Sun, Zhaoliang Yu, Xian Zeng, Rong Xiao, Jason Yongsheng Chan, Peiyong Guan, Yali Wang, Peili Wang, Lizhen Liu, Shijun Wen, Qiang Yu, Choon Kiat Ong, Bin-Tean Teh, Ying Xiong, Jing Tan

**Affiliations:** 1 State Key Laboratory of Oncology in South China, Sun Yat-sen University Cancer Center, Guangzhou, 510060 P. R. China; 2 State Key Laboratory of Oncology in South China, Collaborative Innovation Center for Cancer Medicine, Sun Yat-sen University Cancer Center, 651 East Dongfeng Road, Guangzhou, Guangdong 510060 P. R. China; 3 Cancer and Stem Cell Biology Program, Duke-NUS Medical School, Singapore, Singapore; 4 Department of Oncology, Guangdong Provincial People’s Hospital (Guangdong Academy of Medical Sciences), Southern Medical University, Guangdong, 510060 P. R. China; 5 Department of Laboratory Medicine, Guangzhou First People’s Hospital, School of Medicine, South China University of Technology, Guangdong, 510060 P. R. China; 6 Department of Colorectal Surgery, The Sixth Affiliated Hospital, Sun Yat-sen University, Guangzhou, Guangdong 510655 P. R. China; 7 Laboratory of Cancer Epigenome, Division of Medical Sciences, National Cancer Centre Singapore, Singapore, Singapore; 8 Genome Institute of Singapore, A*STAR, Singapore, Singapore; 9 Center of Medical Research, Guangdong Provincial People’s Hospital (Guangdong Academy of Medical Sciences), Southern Medical University, Guangdong, 510060 P. R. China; 10 Lymphoma Genomic Translational Research Laboratory, Cellular and Molecular Research, National Cancer Centre Singapore, Singapore, Singapore

**Keywords:** Ovarian cancer, EZH2, IDH2, TCA cycle, Metabolic rewiring

## Abstract

**Background:**

Enhancer of zeste homolog 2 (EZH2), the key catalytic subunit of polycomb repressive complex 2 (PRC2), is overexpressed and plays an oncogenic role in various cancers through catalysis-dependent or catalysis-independent pathways. However, the related mechanisms contributing to ovarian cancer (OC) are not well understood.

**Methods:**

The levels of EZH2 and H3K27me3 were evaluated in 105 OC patients by immunohistochemistry (IHC) staining, and these patients were stratified based on these levels. Canonical and noncanonical binding sites of EZH2 were defined by chromatin immunoprecipitation sequencing (ChIP-Seq). The EZH2 solo targets were obtained by integrative analysis of ChIP-Seq and RNA sequencing data. In vitro and in vivo experiments were performed to determine the role of EZH2 in OC growth.

**Results:**

We showed that a subgroup of OC patients with high EZH2 expression but low H3K27me3 exhibited the worst prognosis, with limited therapeutic options. We demonstrated that induction of EZH2 degradation but not catalytic inhibition profoundly blocked OC cell proliferation and tumorigenicity in vitro and in vivo. Integrative analysis of genome-wide chromatin and transcriptome profiles revealed extensive EZH2 occupancy not only at genomic loci marked by H3K27me3 but also at promoters independent of PRC2, indicating a noncanonical role of EZH2 in OC. Mechanistically, EZH2 transcriptionally upregulated *IDH2* to potentiate metabolic rewiring by enhancing tricarboxylic acid cycle (TCA cycle) activity, which contributed to the growth of OC.

**Conclusions:**

These data reveal a novel oncogenic role of EZH2 in OC and identify potential therapeutic strategies for OC by targeting the noncatalytic activity of EZH2.

**Supplementary Information:**

The online version contains supplementary material available at 10.1186/s12943-023-01786-y.

## Background

Ovarian cancer (OC) is among the deadliest cancers worldwide [1]. Most of the OC patients are diagnosed at advanced stages with poor prognosis and limited therapeutic options [2]. Cytoreductive surgery followed by platinum-based chemotherapy remains the standard of care for first-line treatment of OC patients. Even though most patients initially respond to treatment, the high frequency of resistance and relapse hinder the success of contemporary anticancer therapies in OC patients, making OC the leading cause of death in women with gynecological malignancies [3]. Multiple newer therapies, such as antiangiogenic agents, PARP inhibitors and immune checkpoint inhibitors, have been utilized clinically, leading to incremental albeit modest survival improvements in patients with OC. Moreover, the side effects and limited cost-effectiveness of these agents, along with drug resistance, are obstacles for their utilization [4]. Therefore, there is an urgent need to explore novel therapeutics for OC treatment.

DNA copy number alterations are prevalent in OC and drive ovarian tumorigenesis [5]. *EZH2* is an oncogenic driver gene with high copy number amplification [6]. Previous studies have shown that EZH2 is overexpressed in OC, which promotes cell proliferation and invasion, inhibits apoptosis and enhances angiogenesis [7, 8]. These studies in OC mainly focused on the catalytic function of EZH2 via its SET domain methyltransferase activity, which confers trimethylation of histone H3 at lysine 27 (H3K27me3) to suppress gene expression [9]. It has been reported that EZH2 overexpression is associated with poor prognosis in OC and that EZH2 is positively correlated with the level of H3K27me3 in OC [10, 11]. However, other reports show the loss of H3K27me3 in OC patients, which is correlated with poor prognosis [12]. These contradictory findings have raised questions about whether EZH2 plays its oncogenic role in OC solely through its H3K27me3 methyltransferase activity, highlighting the need to further clarify the oncogenic mechanisms of EZH2 in OC.

EZH2 functions as an important chromatin modifier; however, increasing evidence suggests that EZH2 can also act as a transcriptional activator [13, 14]. For example, EZH2 directly transactivates *CCND1* expression to confer a growth advantage independent of its histone methyltransferase activity in natural killer/T-cell lymphoma (NKTL) [15]. In prostate cancer, EZH2 promotes progression via its dual roles: its canonical role in epigenetic silencing of tumor suppressor genes as well as its noncanonical role as a transcription factor in activating AR transcription [16, 17]. Overexpression of EZH2 occurs frequently in various solid tumors and is correlated with poor prognosis, suggesting that there is a compelling rationale for targeting EZH2 in cancer [18, 19]. Currently, several EZH2 inhibitors have been developed for the treatment of hematological malignancies, prostate cancer, sarcoma, and other advanced solid tumors. However, most of these agents are still in preclinical or early-phase clinical trials [20]. In addition, current EZH2 inhibitors mainly suppress its catalytic methylation activity and thus may have therapeutic limitations due to the prevalent roles of noncatalytic functions of EZH2 in tumorigenesis. Taken together, these observations indicate that the ability to harness the antitumor effect of EZH2 inhibitors in OC will remain a clinical challenge, until the mechanisms of EZH2 in OC have been determined.

In this study, we showed that small molecule-induced degradation, rather than catalytic inhibition, of EZH2 significantly blocked OC growth in vitro and in vivo. We demonstrated that EZH2 acts to promote OC in a catalysis-independent manner. We further elucidated that EZH2 exerts this effect by transcriptionally regulating *IDH2* expression, resulting in metabolic rewiring. Finally, we identified a subset of patients with OC showing higher expression of EZH2 but a lower H3K27me3 level, which conferred the worst prognosis among OC patients. This study revealed a noncanonical role of EZH2 in OC and demonstrated that targeting the catalysis-independent activity of EZH2 is a potential therapeutic strategy.

## Methods

Detailed procedures were provided in Supplemental Methods.

### Clinical samples, cell lines, and reagents

Informed consent was obtained from the patients who provided the tissue samples, and all procedures were approved by the medical ethics committee of the Sun Yat-sen University Cancer Center (Guangzhou, China). All commercial cell lines were purchased from ATCC, except for COV504 (obtained from ECACC). Authentication of cell lines was performed by the authors. The cell lines were maintained in RPMI 1640 medium (HyClone) supplemented with 10% fetal bovine serum (HyClone) and 1% penicillin/streptomycin (Gibco). Mycoplasma testing was performed using the MycoSensor PCR assay kit (Stratagene). DZNep (S7120), GSK126 (S7061) and EPZ-6438 (S7128) were purchased from Selleck Chemicals. YM281 was a kind gift from Dr. S. Wen (Sun Yat-sen University Cancer Center, Guangzhou, China).

### In vivo studies

All animal studies were conducted in compliance with animal protocols approved by the Institutional Animal Care and Use Committee of Sun Yat-sen University Cancer Center (Guangzhou, China). Female BALB/c nude mice (5–6 weeks old) were purchased from Beijing Vital River Laboratory Animal Technology Company. Tumors were measured with Vernier calipers, and the tumor volume was calculated with the following formula: tumor volume = length × width^2^/2. When the tumor volume reached approximately 100 mm^3^, the mice were divided into different groups for treatment. Randomization was performed by equally dividing the tumor-bearing mice with a similar tumor burden into groups for drug treatment. For the EZH2-inducible knockdown xenograft assay, mice were implanted subcutaneously in the flank with 2 × 10^6^ OVCAR8 cells with inducible sh-scramble (shSCR) or sh*EZH2* expression. Doxycycline was dissolved in distilled water and administered by oral gavage at 150 mg/kg daily. For the treatment with DZNep and EPZ-6438, mice were implanted subcutaneously in the right flank with 5 × 10^6^ OVCAR8 cells. For the PDX models, PDX-POVC15 tumor masses were passaged in NOD/SCID mice after subcutaneous implantation. When the tumor volumes reached approximately 100 mm^3^, the mice were randomly divided into three groups. DZNep was suspended in 1× saline and was given twice a week by intraperitoneal injection (1 mg/kg). EPZ-6438 was dissolved in vehicle consisting of 0.5% weight/volume sodium carboxymethylcellulose and 0.1% volume/volume Tween 80 and given by oral gavage (40 mg/kg daily). For the treatment with YM281, mice were administered vehicle control (80% PBS, 10% castor oil, and 10% DMSO) or YM281 (80 mg/kg) through intraperitoneal injection 6 times weekly. Tumor volume and body weight were monitored twice a week until the tumor volume reached 1,000 mm^3^. Mice were sacrificed by CO_2_ inhalation, and tumors were harvested for further analysis.

### Statistical analysis

The data are presented as the mean ± SD values unless otherwise specified. Significant differences between two groups were identified using two-tailed Student’s t test for unpaired data, and *P* < 0.05 was considered to indicate a statistically significant difference. All statistical analyses were performed by GraphPad Prism 9 (La Jolla, CA).

## Results

### Overexpression of EZH2 combined with a low H3K27me3 level correlates with the worst prognosis in patients with OC

**Fig. 1:**
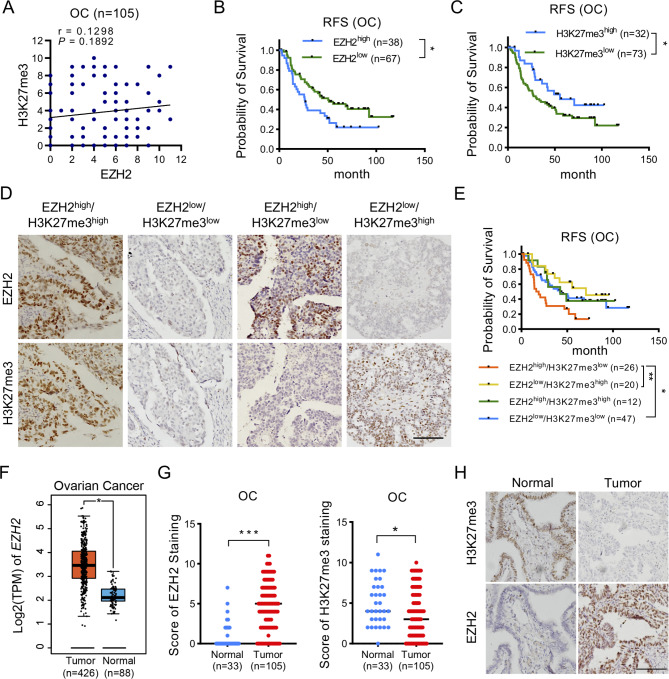
Overexpression of EZH2 with low H3K27me3 correlates with the worst prognosis in patients with OC (**A**) Correlation analysis between EZH2 and H3K27me3 levels in the OC cohort (n = 105). *P* value was determined by Pearson’s correlation test (**B**) Kaplan-Meier survival analysis for the OC cohort (n = 105), which was divided into EZH2^high^ and EZH2^low^ subgroups based on the cutoff (cutoff = 6) calculated by the X-Tile software. The *P* value was calculated by log-rank test. *P < 0.05 (**C**) Kaplan-Meier survival analysis for the OC cohort (n = 105), which was divided into H3K27me3^high^ and H3K27me3^low^ subgroups based on the cutoff (cutoff = 6) calculated by the X-Tile software. The *P* value was calculated by log-rank test. *P < 0.05 (**D**) Representative IHC images of EZH2 and H3K27me3. EZH2 and H3K27me3 levels were detected by IHC staining in a cohort of 105 patients with OC. Scale bar: 100 μm (**E**) Kaplan-Meier survival analysis for OC samples (n = 105) in four subgroups, which was classified based on EZH2 (cutoff = 6) and H3K27me3 (cutoff = 6) levels. The *P* value was calculated by log-rank test. *P < 0.05, **P < 0.01 (**F**) EZH2 log2(TPM) value in normal ovarian tissues (GTEx, n = 88) and ovarian cancer tissues (TCGA, n = 426). Data are presented as the mean ± SEM. *P < 0.05 (**G**) Staining score of EZH2 and H3K27me3 levels in fallopian tube and OC tissues. IHC score of EZH2 and H3K27me3 was determined as the scores for the proportion of positively stained tumor cells (1, 1–25%; 2, 26–50%; 3, 51–75%; 4, > 75%) and staining intensity (0, no staining; 1, weak; 2, moderate; 3, strong) by each investigator were multiplied and then averaged. *P < 0.05, ***P < 0.0001 (**H**) Representative images of EZH2 and H3K27me3 staining in fallopian tube (n = 33) and OC (n = 105) tissues. Scale bar: 100 μm

To investigate the clinical significance of EZH2 and H3K27me3 in OC, we examined the protein level of EZH2 and the level of H3K27me3 in a cohort consisting of 105 OC patients using an IHC approach. The IHC data indicated that the EZH2 and H3K27me3 levels were not significantly correlated in our cohort of OC patients **(**Fig. 1A**)**. Patients with high levels of EZH2 exhibited a worse prognosis than those with low levels of EZH2, whereas high levels of H3K27me3 predicted a better prognosis in OC patients **(**Fig. 1B and C**)**. Based on the patterns of EZH2 expression and the H3K27me3 level, we classified OC patients into four subgroups: EHZ2^high^/H3K27me3^high^ (11%), EHZ2^low^/H3K27me3^low^ (45%), EHZ2^low^/H3K27me3^high^ (19%) and EHZ2^high^/H3K27me3^low^ (25%) **(**Fig. 1D**)**. In addition, after stratification of patients by H3K27me3 levels, the EZH2^low^/H3K27me3^high^ pattern correlated with the better survival outcomes, and patients in the EZH2^high^/H3K27me3^high^ subgroup still had a worse prognosis **(**Fig. 1E**)**. These observations are consistent with a previous study showing that overexpression of EZH2 with distinct level patterns of H3K27me3 levels correlates with different prognoses in patients with OC, suggesting a decoupling of EZH2 and H3K27me3 in OC tumorigenesis [21]. Furthermore, we showed that in both The Cancer Genome Atlas (TCGA) and Genotype-Tissue Expression (GTEx) projects [22] and in our own in-house data, EZH2 was overexpressed in OC tissues compared to the normal tissues, whereas the H3K27me3 level was decreased in OC tissues compared to normal fallopian tube tissues **(**Fig. 1F-H**)**. Together, these results suggest that the oncogenic role of EZH2 may be independently of its catalytic activity in OC.

### EZH2 functions as an oncogenic driver to promote OC growth independently of its catalytic activity

**Fig. 2:**
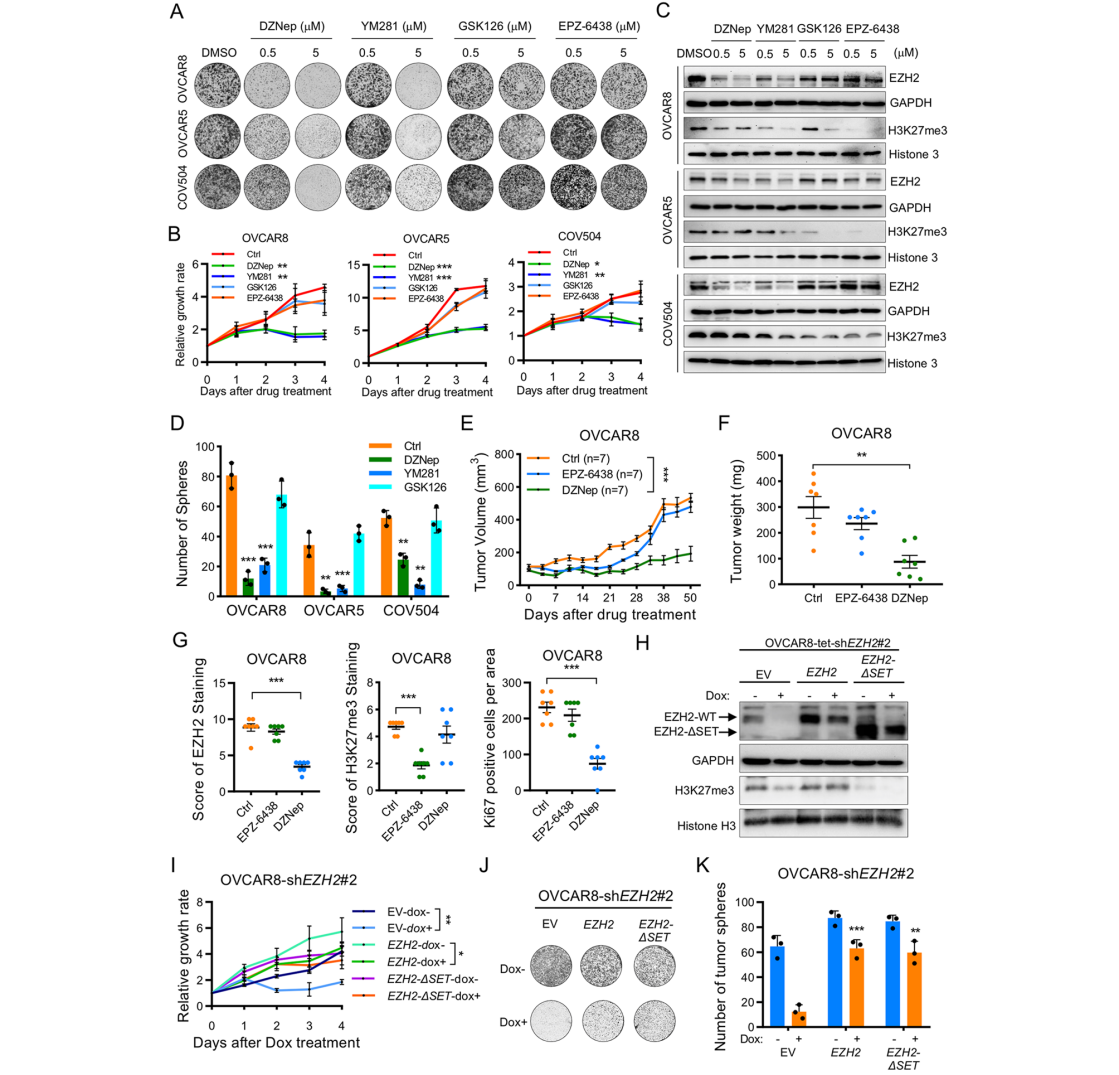
EZH2 functions as an oncogenic driver to promote OC growth independently of its catalytic activity (**A**) Colony formation assay in three OC cell lines. Cells were treated with DMSO or four different EZH2 inhibitors with indicated concentrations for 12 days. Growth medium was changed and inhibitors were also replenished every 3 days. Images are representative of three independent experiments **(B)** Cell growth curve of 3 OC cell lines treated with DZNep (1µM), YM281 (5µM), GSK126 (5µM) or EPZ-6438 (5µM) for 96 h. Data are presented as the mean ± SD. *P < 0.05, **P < 0.01, ***P < 0.001 (**C**) Immunoblotting analysis of EZH2 and H3K27me3 in 3 OC cell lines treated with four different EZH2 inhibitors with indicated concentrations (**D**) Tumorsphere formation assay of OC cells treated with DZNep (1µM), YM281 (5µM), GSK126 (5µM) for 10–12 days. Data are presented as the mean ± SD. **P < 0.01, ***P < 0.001 (**E-F**) Xenograft tumor growth (E) and tumor weight (F) of OVCAR8 cells in nude mice treated with saline as control, EPZ-6438 at 200 mg/kg intragastrically daily, or DZNep at 1 mg/kg intraperitoneally twice a week. n = 7 per group. Data are presented as mean ± SEM. **P < 0.01, ***P < 0.001(Two-way ANOVA with Tukey’s post hoc test) (**G**) IHC analysis of EZH2, H3K27me3, and Ki67 levels in the excised tumors from (F). Data are presented as mean ± SEM. ***P < 0.001 (**H**) Immunoblot analysis of EZH2 and H3K27me3 levels in sh*EZH2*-inducible OVCAR8 cell lines with wildtype or mutant EZH2 restoration. Expression of EZH2-WT and the EZH2-ΔSET was detected by an EZH2 antibody against residues surrounding Arg354 of human EZH2 protein (SET domain locates in 612–727 amino acids). GAPDH and Histone H3 were used as loading controls (**I**) Cell growth curve of sh*EZH2*-inducible OVCAR8 cell lines with wildtype or mutant EZH2 restoration in the presence or absence of Dox at 1 µg/mL for 96 h. Data are presented as mean ± SD. ***P < 0.001 (**J**) Colony formation assay in sh*EZH2*-inducible OVCAR8 cell lines with wildtype or mutant EZH2 restoration in the presence or absence of 10ng/mL Dox for 12 days. Growth medium was changed and doxycycline was also replenished every 3 days. Images are representative of three independent experiments (**K**) Tumorsphere formation assay of indicated cells treated with or without Dox at 10ng/mL for 10 days. Data are presented as the mean ± SD. **P < 0.01, ***P < 0.001 vs. EV groups

To investigate the function of EZH2 in OC, we applied 4 inhibitors of EZH2 in three OC cell lines that show high expression of EZH2. The first EZH2 inhibitor described, DZNep has been reported to induce EZH2 degradation and inhibit histone methylation [23]. YM281 was recently reported as a small molecule EZH2 degrader [24]. GSK126 and EPZ-6438 are catalytic inhibitors of EZH2 that bind to its SET domain and compete with the cofactor S-adenosylmethionine (SAM) without affecting the protein stability of EZH2 [20]. Intriguingly, in both the clonogenic growth and cell proliferation assays, DZNep and YM281 but not GSK126 or EPZ-6438 suppressed the growth of OC cell lines **(**Fig. 2A and B, Supplementary Figure S1A). Using immunoblotting assay, we observed that both DZNep and YM281 could reduce the levels of EZH2 and H3K27me3, whereas GSK126 and EPZ-6438 could decrease only the level of H3K27me3 and did not affect the EZH2 protein level **(**Fig. 2C**)**. These results suggest that the oncogenic function of EZH2 in OC is independent of its catalytic activity. Consistent with these findings, we observed that DZNep and YM281 but not GSK126 attenuated the capacity of tumor sphere formation **(**Fig. 2D and Supplementary Figure S1B). The differential efficacies of DZNep, YM281 and EPZ-6438 were further demonstrated in a xenograft model. After continuous dosing with DZNep and EPZ-6438, we found that DZNep but not EPZ-6438 significantly suppressed tumor growth in vivo **(**Fig. 2E-F**)**. In DZNep-treated tumors, the Ki67 level was decreased concomitantly with the EZH2 but not the H3K27me3 level, further supporting the idea that the oncogenic role of EZH2 is independent of its catalytic methylation activity **(**Fig. 2G and Supplementary Figure S1C). These results revealed that a reduction in the level of EZH2 but not inhibition of its catalytic activity suppresses the progression of OC.

Since DZNep and YM281 suppressed the proliferation of OC cells by reducing the EZH2 level, the effect of EZH2 depletion in OC was investigated via a doxycycline-inducible Tet-On system expressing EZH2 short hairpin RNA (shRNA). The addition of doxycycline effectively induced the expression of EZH2 shRNA, which drastically decreased the level of EZH2 in three OC cell lines (Supplementary Figure S1D). EZH2 depletion not only suppressed cell proliferation but also attenuated the capacity of tumor sphere formation in OC cells (Supplementary Figure S1E and F). The oncogenic effect of EZH2 was further validated in vivo using OVCAR8 cells harboring the same system; downregulation of EZH2 expression following doxycycline treatment significantly inhibited xenograft tumor growth (Supplementary Figure S1G and H). These results provide strong evidence that EZH2 functions as a bona fide oncogene in OC.

To determine whether the catalytic activity of the SET domain is required for the oncogenic function of EZH2, we generated OVCAR8 and OVCAR5 cells stably expressing EZH2 or EZH2-ΔSET (lacking the SET domain) with the EZH2 inducible knockdown construct sh*EZH2*#2. We introduced a silent point mutation at the *EZH2*#2 shRNA target site in the cloned *EZH2* and *EZH2-ΔSET* gene sequences, which enabled us to study the differential effect of endogenous and ectopic gene expression. As expected, ectopic expression of EZH2 but not the EZH2-ΔSET restored the H3K27me3 level, which was reduced upon EZH2 silencing **(**Fig. 2H and Supplementary Figure S1I). However, both *EZH2* and *EZH2-ΔSET* were able to rescue the suppressed tumor growth and tumor sphere formation induced by EZH2 silencing **(**Fig. 2I-K and Supplementary Figure S1J-H). Collectively, these results demonstrate that EZH2 promotes OC initiation and proliferation independently of its catalytic activity in OC.

### EZH2 noncanonically occupies promoters associated with metabolic genes

**Fig. 3:**
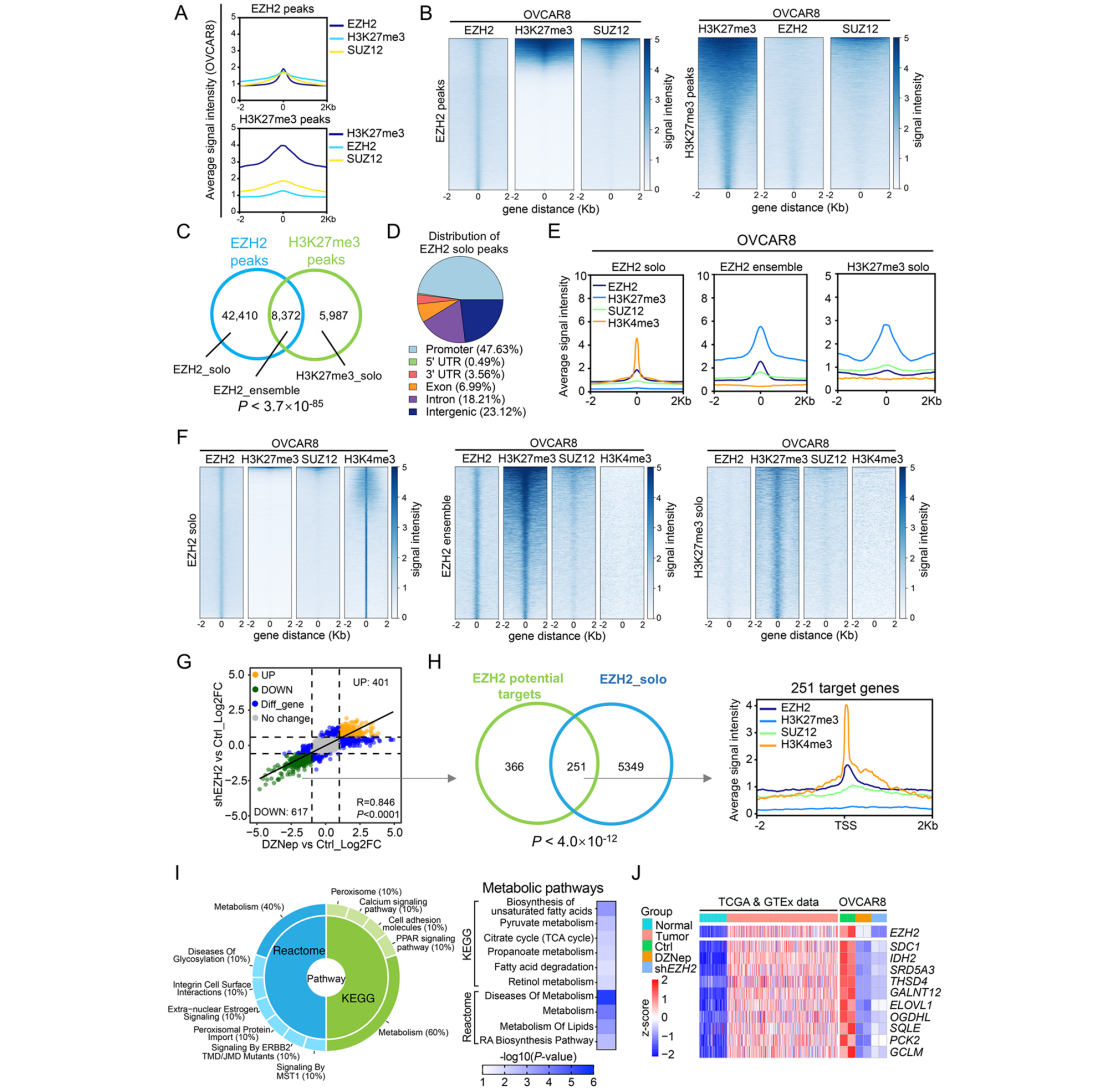
EZH2 noncanonically occupies promoters associated with metabolic genes (**A-B**) Averaged signal intensities (A) and heatmaps (B) for EZH2, H3K27me3 and SUZ12 ± 2 kb from the centres of EZH2 or H3K27me3 peaks in OVCAR8 cells (**C**) Venn diagram showing the significant overlap between EZH2 and H3K27me3 binding sites identified in OVCAR8 cells (**D**) Pie-chart plot showing the genomic distribution of peaks with EZH2-solo binding in OVCAR8 cells (**E-F**) Averaged signal intensities (E) and heatmaps (F) for EZH2, H3K27me3, SUZ12 and H3K4me3 ± 2 kb from the centres of EZH2-solo, EZH2-ensemble or H3K27me3-solo peaks in OVCAR8 cells (**G**) Scatterplot showing log2 fold change of genes expression in OVCAR8 cells after DZNep treatment or doxycycline-induced EZH2 silencing. Significant differential expression genes (DEGs) upon either treatments are indicated in blue color, upregulated DEGs in both groups are indicated in orange color, downregulated DEGs in both groups are indicated in green color. Spearman correlation between all genes is shown as a black line (R = 0.846, p < 0.0001) (**H**) Venn diagram showing the significant overlap between 617 EZH2 potential targets and EZH2-solo peaks associated genes in OVCAR8 cells. Averaged signal intensities for EZH2, H3K27me3, SUZ12 and H3K4me3 ± 2 kb from the TSS of overlap genes are shown (**I**) Gene set enrichment analysis for the 251 EZH2 solo targets using the KEGG and Reactome gene sets. Metabolism-related pathways are among the most enriched (left). The -log10(*P*-value) of metabolism-related pathways are shown (right) (**J**) Metabolism-related genes are upregulated in ovarian cancer tissues, and down regulated after EZH2 reduction. A heatmap showing the relative expression of top 10 metabolic genes in (H) by calculating their Z-score in indicated RNA-Seq data

To systematically investigate the gene-regulatory roles of EZH2 in OC, we carried out integrative analysis of ChIP-Seq data to profile the genome-wide occupancy of EZH2, H3K27me3 and SUZ12, a subunit in the PRC2 complex, in OVCAR8 cells. A large majority of EZH2 peaks lacked H3K27me3 and SUZ12 binding and were termed as EZH2-solo binding sites, and only a small subset of EZH2 peaks overlapped with H3K27me3 and SUZ12 peaks, specifying canonical EZH2-ensemble binding sites (Fig. 3A-C). Approximately 50% of the EZH2-solo peaks were located at promoters (Fig. 3D). To confirm that these EZH2 solo binding sites are active promoters, an additional chromatin mark H3K4me3 was analyzed. Compared to the EZH2 ensemble and H3K27me3 solo binding sites, the EZH2 solo binding sites were overwhelmingly enriched in the H3K4me3 peaks (Fig. 3E **and F**). These results demonstrate that EZH2 solo binding sites are prevalent in OC and potentially regulate gene activation, differing from the canonical roles of the PRC2 complex.

To define the EZH2 solo target genes in OC, we performed RNA-Seq analysis in OVCAR8 cells upon DZNep treatment or silencing of EZH2. The transcriptional changes occurring in response to the two different approaches of EZH2 depletion were highly correlated (Fig. 3G). DZNep treatment resulted in upregulation of 833 genes and downregulation of 850 genes, while silencing of EZH2 caused upregulation of 1007 genes and downregulation of 1127 genes (Fig. 3G). Since the noncanonical function of EZH2 is to activate gene expression, genes downregulated upon EZH2 reduction were regarded as potential EZH2 potential targets. The 617 overlapping downregulated genes were further intersected with the EZH2 solo peak genes, and the resulting 251 overlapping genes enriched in EZH2 and H3K4me3 peaks but not H3K27me3 or SUZ12 peaks were defined as EZH2 solo targets (Fig. 3H). Gene set enrichment analysis (GSEA) using the KEGG and Reactome databases revealed that the most significantly negatively enriched pathways upon EZH2 depletion were metabolic signaling pathways **(**Fig. 3I**)**, suggesting a role of EZH2 in metabolic regulation in OC. Furthermore, we identified 57 EZH2 solo targets contributing to the metabolic signaling pathways overlapping between the DZNep treatment and EZH2 silencing groups. The expression profiles of these 57 metabolic genes were further verified in normal ovarian and ovarian tumor tissues from the TCGA cohort and GTEx project. Twelve of these 57 genes were upregulated (log2foldchange > 1, p-value < 0.05) in ovarian cancer **(**Fig. 3J**)**. Additionally, these 12 genes were downregulated upon both pharmacological and genetic EZH2 depletion, implying that EZH2 plays its oncogenic role partially via these genes **(**Fig. 3J**)**. Taken together, these results indicate that the noncanonical roles of EZH2 in OC might be associated with metabolic rewiring.

### EZH2 transcriptionally upregulates ***IDH2*** to promote metabolic rewiring

**Fig. 4:**
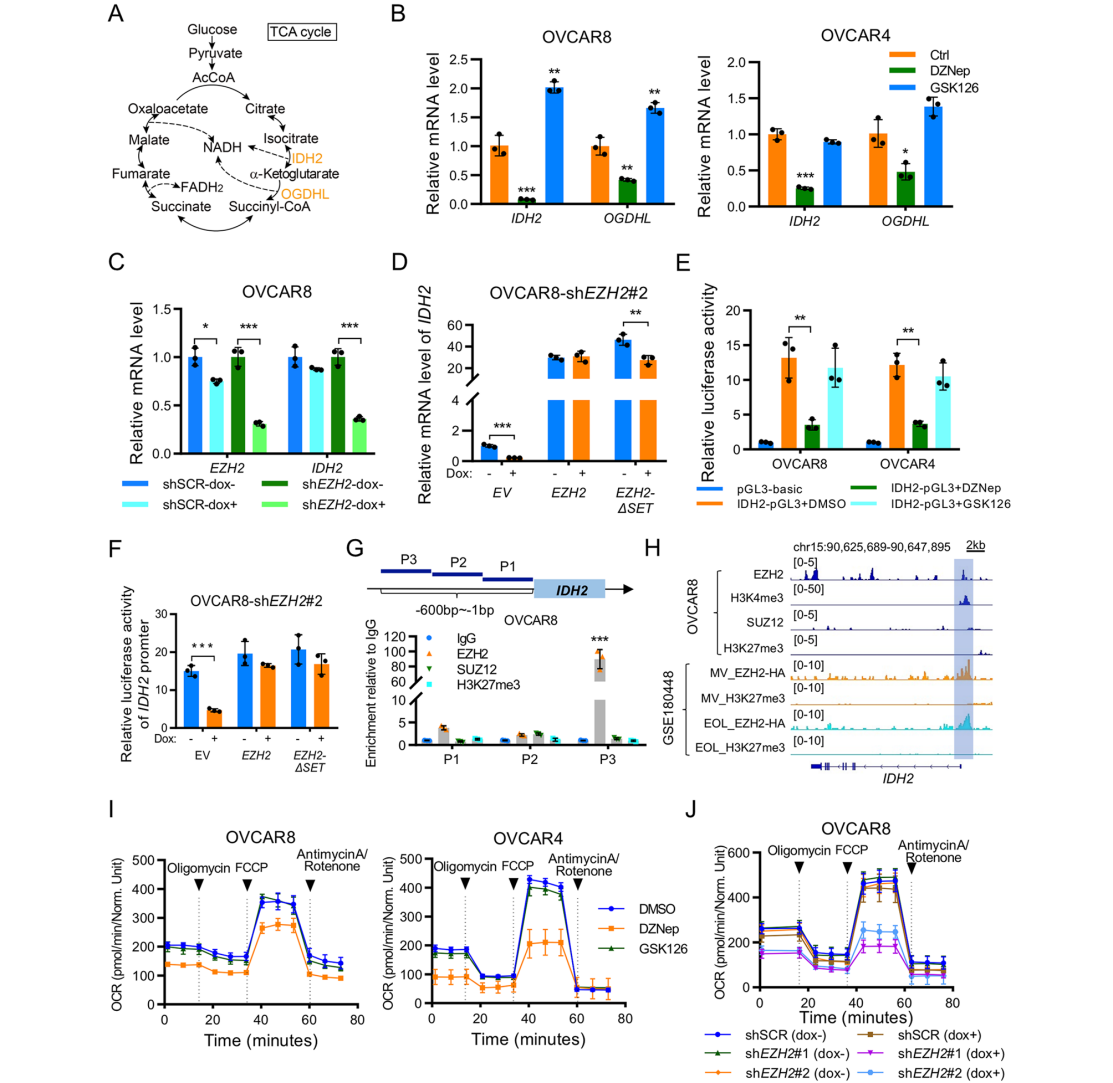
EZH2 transcriptionally upregulates *IDH2* to promote metabolic rewiring (**A**) Diagram of TCA cycle. TCA cycle related genes in Fig. 3I are indicated in orange color (**B**) qRT-PCR analysis of TCA cycle related genes in OVCAR8 and OVCAR4 cells upon 5µM DZNep or GSK126 treatment for 72 h. Data are presented as mean ± SD. *P < 0.05, **P < 0.01, ***P < 0.001 (**C-D**) qRT-PCR analysis of EZH2 and IDH2 levels upon Dox-induced EZH2 silencing in indicated cells. IDH2 level was concomitantly reduced upon Dox-induced EZH2 silencing (C), and ectopic expression of *EZH2* or *EZH2-ΔSET* restored the level of IDH2 (D). Data are presented as mean ± SD. *P < 0.05, **P < 0.001, ***P < 0.001 (**E-F**) Dual-luciferase reporter assay of *IDH2* promoter activity in OC cell lines upon pharmacological or genetic inhibition of EZH2. The OVCAR8 and OVCAR4 cells were treated with 5µM DZNep or GSK126 for 48 h before luciferase reporter assay (E). The indicated cells were treated with 1 µg/mL Dox for 48 h before luciferase reporter assay (F). Data are presented as mean ± SD. **P < 0.01, ***P < 0.001 (**G**) ChIP assay in OVCAR8 cells. A specific antibody against EZH2 but not isotype IgG, SUZ12, or H3K27me3 could capture the fragment containing EZH2 binding site in the *IDH2* promoter region, which was amplified by specific primers (top) by qRT-PCR (bottom) (**H**) ChIP-seq profiling showed the ChIP-seq signal for EZH2, H3K4me3, H3K27me3 and SUZ12 at the genomic loci of *IDH2* in OVCAR8 cells. ChIP-seq profiling also showed the ChIP-seq signal for EZH2 and H3K27me3 in two MLL1-rearranged leukaemia cell lines in GSE180448. (**I-J**) OCR assays upon pharmacological (I) or genetic (J) inhibition of EZH2. Representative OCR pattern as a function of time (in min), normalized to total protein levels. Cells were pretreated with 5 µM DZNep, GSK126 or 1 µg/mL doxycycline for 72 h before OCR measurement. Data are shown as mean ± SD of three replicates per treatment

OC was previously found to exhibit metabolic heterogeneity, and OXPHOS is one of the major mechanisms of energy production in OC cells [25]. Interestingly, the genes encoding isocitrate dehydrogenase 2 (IDH2) and oxoglutarate dehydrogenase L (OGDHL), which participate in oxidative phosphorylation (OXPHOS) and the tricarboxylic acid cycle (TCA cycle), were among the top 10 genes upregulated when EZH2 was inhibited **(**Fig. 3J **and** Fig. 4A**)**. In addition, we performed gene expression profiling analysis with the RNA-Seq data from the TCGA ovarian cancer cohort. KEGG pathway analysis showed that the oxidative phosphorylation pathway was significantly enriched in ovarian cancer tissues compared to normal ovarian tissues, suggesting that the TCA cycle or oxidative phosphorylation activity plays an important role in ovarian cancer development (**Supplementary Figure S2A**). These results raised the possibility that EZH2 is involved in the mitochondrial TCA cycle to promote OXPHOS in OC.

To determine whether EZH2 promotes TCA cycle activity, we next examined the mRNA levels of *IDH2* and *OGDHL* upon DZNep or GSK126 treatment in OVCAR8 and OVCAR4 cells. Interestingly, *IDH2* and *OGDHL* were downregulated upon treatment with DZNep but not GSK126, suggesting that EZH2 regulates the TCA cycle independently of its methyltransferase activity **(**Fig. 4B**)**. We showed that IDH2, the rate-limiting enzyme for the TCA cycle, is significantly upregulated in OC, which prompted us to investigate the correlation between EZH2 and IDH2. We observed that the IDH2 level was concomitantly reduced upon doxycycline-induced EZH2 silencing **(**Fig. 4C**)**, and that ectopic expression of *EZH2* or *EZH2-ΔSET* restored the level of IDH2 **(**Fig. 4D**)**, indicating that *IDH2* might be transcriptionally regulated by EZH2. In addition, we further inserted the promoter and 5’UTR of *IDH2* (-1000 ~ 81 bp) into the pGL3-basic plasmid and performed a dual luciferase reporter assay after DZNep and GSK126 treatment in OVCAR8 and OVCAR4 cells. The transcriptional activity of the *IDH2* promoter was attenuated upon treatment with DZNep but not GSK126 **(**Fig. 4E**)**. Furthermore, we performed a dual luciferase reporter assay in OVCAR8-sh*EZH2*#2 cells transduced with the *EZH2-* or *EZH2-ΔSET-*expressing plasmids. Doxycycline-induced EZH2 silencing decreased luciferase activity driven by the *IDH2* promoter, whereas ectopic expression of *EZH2* and *EZH2-ΔSET* restored *IDH2* promoter activity **(**Fig. 4F**)**. Moreover, we examined the binding regions of EZH2, SUZ12 and H3K27me3 in the *IDH2* promoter by chromatin immunoprecipitation (ChIP). Compared with the IgG isotype control, the specific anti-EZH2 antibody but not the specific anti-SUZ12 or anti-H3K27me3 antibody bound directly to the region containing − 600~-400 bp in the *IDH2* promoter **(**Fig. 4G**)**. In addition, in our ChIP-Seq data and the GSE180448 data, the binding peaks of EZH2 but not those of H3K27me3 or SUZ12 were located in the promoter region of *IDH2*, providing further evidence supporting our hypothesis that EZH2 transcriptionally upregulates *IDH2* via a noncatalytic function (Fig. 4H) [17, 26]. These results demonstrated that EZH2 serves as a direct transcriptional regulator to promote *IDH2* expression in OC.

To confirm the regulatory effect of EZH2 on OXPHOS activity, we further examined the oxygen consumption rate (OCR) in OVCAR8 cells upon DZNep and GSK126 treatment. The basal and maximal OCRs were decreased upon treatment with DZNep but not GSK126 in OVCAR8 and OVCAR4 cells **(**Fig. 4I**).** Consistent with these findings, doxycycline induced *EZH2* silencing also resulted in significant reductions in the basal and maximal OCRs in OVCAR8 cells **(**Fig. 4J**)**. Furthermore, we demonstrated that the OCR of cells was restored after genetic knockdown of EZH2 and restoration with wild-type or mutant EZH2 (**Supplementary Figure S2B**). These data support a role for EZH2 in the regulation of OXPHOS in OC cells.

### Reduction of EZH2 impairs IDH2 dependent metabolic rewiring to suppress OC growth

**Fig. 5:**
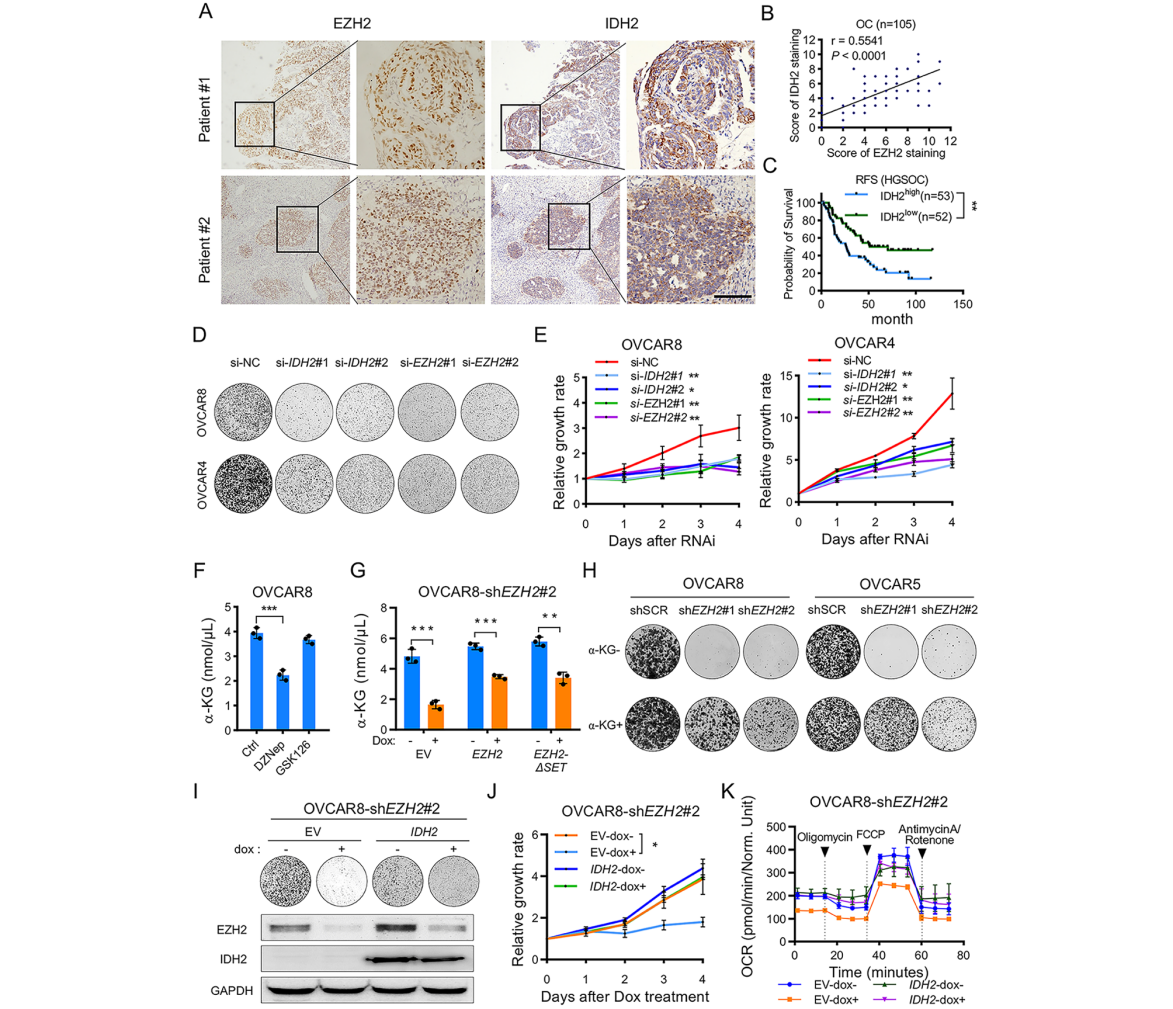
Reduction of EZH2 impairs IDH2 dependent metabolic rewiring to suppress OC growth (**A**) Representative images of EZH2 and IDH2 staining in OC tissues (n = 105). Scale bar: 100 μm (**B**) IDH2 level was strongly positively correlated with EZH2 level in the OC cohort (n = 105). *P* value was determined by Pearson’s correlation test (**C**) Kaplan-Meier survival analysis for the OC cohort (n = 105), which was divided into IDH2^high^ and IDH2^low^ subgroups based on the cutoff (cutoff = 5) calculated by the X-Tile software. The *P* value was calculated by log-rank test. **P < 0.01 (**D**) Colony formation assay in OVCAR8 and OVCAR4 cells transfected with *IDH2* or *EZH2* siRNA. Cells were cultured for 12 days. Growth medium was changed every 3 days. Images are representative of three independent experiments (**E**) Cell growth curve of OVCAR8 and OVCAR4 cells transfected with *IDH2* or *EZH2* siRNA for 96 h. Data are presented as mean ± SD. *P < 0.05, **P < 0.01 (**F-G**) α-KG assay in OVCAR8 cells upon DNZep, GSK126 treatment (F) or in OVCAR8-sh*EZH2*#2 cells ectopic expressing wildtype or mutant *EZH2* (G). The OVCAR8 cells were treated with 2µM DZNep, 5µM GSK126 or 1 µg/mL doxycycline for 72 h before α-KG assay. Data are presented as the mean ± SD. **P < 0.01, ***P < 0.001 (**H**) Colony formation assay in OVCAR8 and OVCAR5 cells upon Dox-induced EZH2 silencing with or without α-KG supplementation. Cells were treated with 5ng/mL doxycycline for 12 days. Medium with or without 1mM α-KG was changed and doxycycline was also replenished every 2 days. Images are representative of three independent experiments (**I**) Colony formation assay in OVCAR8-sh*EZH2*#2 cells with ectopic *IDH2* expression. Cells were treated with 5ng/mL doxycycline for 12 days. Growth medium was changed and doxycycline was also replenished every 3 days. Images are representative of three independent experiments. Immunoblot analysis of EZH2 and IDH2 levels in indicated samples. Cells were treated with or without Dox for 72 h before being harvested. GAPDH was used as loading controls (**J**) Cell growth curve of OVCAR8-sh*EZH2*#2 cells with ectopic *IDH2* expression upon 1 µg/mL doxycycline treatment for 96 h. Data are presented as the mean ± SD. *P < 0.05 (**K**) Representative OCR pattern as a function of time (in min), normalized to total protein levels. Cells were incubated in the absence or presence of 1 µg/mL doxycycline for 72 h before OCR measurement. Data are shown as mean ± SD of three replicates per treatment

To investigate whether the downregulation of *IDH*2 mediated by EZH2 is clinically associated with OC, we performed IHC staining in our OC cohort and confirmed that IDH2 expression was positively correlated with EZH2 expression and that high expression of IDH2 predicted poor prognosis in OC patients (Fig. 5A-C). We further examined the colony formation and cell proliferation abilities upon silencing *IDH2* or *EZH2*. Consistent with the above findings, silencing of either *IDH2* or *EZH2* was sufficient to suppress OC cell growth (Fig. 5D **and E**). We next examined the product synthesized via IDH2, α-ketoglutarate (α-KG), in OVCAR8 cells treated with DZNep or GSK126. The data indicated that the level of α-KG was significantly decreased upon treatment with DZNep but not GSK126 (Fig. 5F). We further evaluated the cellular α-KG level in cells after genetic knockdown of EZH2 and replacement with wild-type or mutant EZH2, demonstrating that the α-KG level was partially restored in OVCAR8 cells with Dox-induced EZH2 silencing by ectopic expression of *EZH2* or *EZH2-ΔSET* (Fig. 5G). Moreover, when we supplemented cultures of OVCAR8 and OVCAR5 cells with α-KG after Dox-induced EZH2 silencing, the phenotype induced by EZH2 knockdown was partially rescued (Fig. 5H), suggesting that the oncogenic function of EZH2 in OC could be partially mediated by α-KG.

To examine whether the anticancer effect of EZH2 reduction is IDH2 dependent, we generated OVCAR8 cells stably expressing *IDH2* and containing the EZH2 inducible knockdown construct sh*EZH2*#2. *IDH2* overexpression counterbalanced the downregulation of *IDH2* mediated by doxycycline-induced *EZH2* silencing **(**Fig. 5I**)**. Next, we studied the tumorigenic behaviors of *IDH2*-overexpressing cells upon doxycycline induced *EZH2* silencing. Ectopic expression of *IDH2* partially rescued the reductions in colony formation and cell proliferation occurring upon doxycycline-induced EZH2 silencing **(**Fig. 5I **and J)**. Furthermore, we found that the basal and maximum OCRs were restored in IDH2-overexpressing cells even upon doxycycline induced *EZH2* silencing **(**Fig. 5K**)**. Taken together, these results indicated that a reduction in EZH2 expression impairs IDH2 dependent metabolic rewiring to suppress OC growth.

### Targeting EZH2-mediated metabolic rewiring exhibits potential therapeutic efficacy in preclinical models of OC

**Fig. 6:**
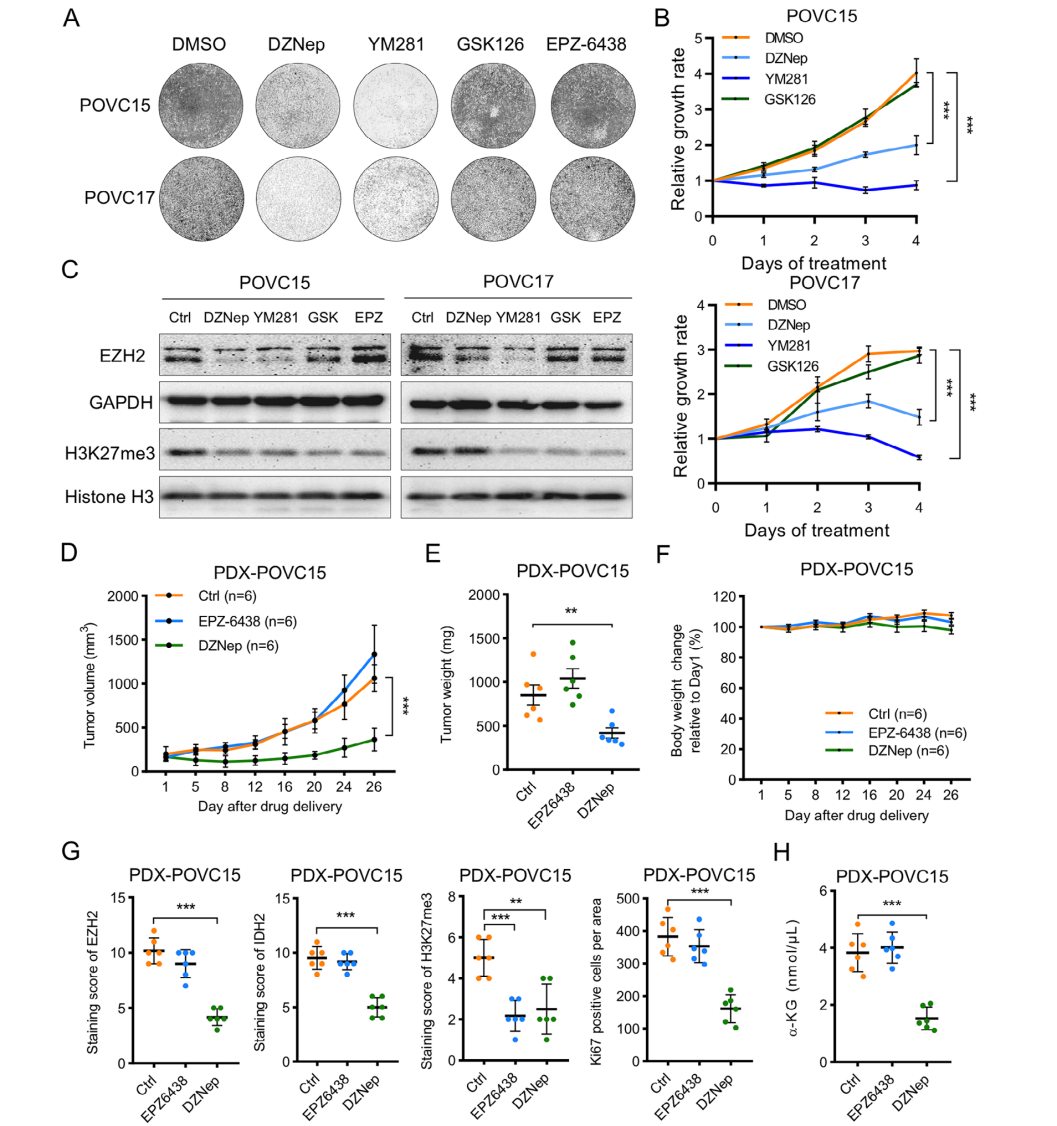
Targeting EZH2 mediated metabolic rewiring exhibits potential therapeutic efficacy in preclinical models of OC (**A**) Colony formation assay in two primary OC cell lines derived from two OC patients (POVC17, POVC15). Cells were treated with DMSO, DZNep (1µM), YM281 (5µM), GSK126 (5µM) or EPZ-6438 (5µM) for 12 days. Growth medium was changed and inhibitors were also replenished every 3 days. Images are representative of three independent experiments unless stated otherwise (**B**) Cell growth curve of two OC patient-derived primary tumor cell lines treated with DMSO, DZNep (1µM), YM281 (5µM) or GSK126 (5µM) for 96 h. Data are presented as the mean ± SD. ***P < 0.001 (**C**) Immunoblotting analysis of EZH2 and H3K27me3 levels in two OC patient-derived primary tumor cell lines upon EZH2 inhibitors treatment. Cells were treated with DMSO, DZNep (1µM), YM281 (5µM), GSK126 (5µM) or EPZ-6438 (5µM) for 72 h **(D)** Tumor growth of PDX models in NOD-SCID mice treated with saline as control, EPZ-6438 at 200 mg/kg intragastrically daily, or DZNep at 1 mg/kg intraperitoneally twice a week (n = 6 per group). Data are presented as mean ± SD. ***P < 0.001(Two-way ANOVA with Tukey’s post hoc test) **(E)** Bar graph showing the tumor weight on the 26th day post-treatment week (n = 6 per group). Data are presented as mean ± SD. **P < 0.01 **(F)** Body weight of each mouse post-treatment. Data are presented as mean ± SD. **(G)** IHC analysis of EZH2, H3K27me3, IDH2 and Ki67 levels in the excised tumors from PDX models. Data are presented as mean ± SD. **P < 0.01, ***P < 0.001 (**H**) α-KG assay in PDX tumors upon EPZ6438 or DZNep treatment. Data are presented as mean ± SD. ***P < 0.001

To further validate the role of EZH2-mediated metabolic rewiring in OC patients, we validated our findings in two primary OC cell lines derived from OC patients. Similarly, only the DZNep and YM281 inhibited the proliferation of patient-derived OC cells. Neither GSK126 nor EPZ-6438 exhibited growth inhibitory effects on in these patient-derived OC cell lines **(**Fig. 6A **and B, Supplementary Figure S3A)**. We found that both DZNep and YM281 could reduce the levels of EZH2 and H3K27me3, whereas GSK126 and EPZ-6438 could only decrease only the level of H3K27me3 and did not affect the EZH2 protein levels in patient-derived OC cells **(**Fig. 6C**)**.

Since chemoresistance has become a major obstacle in the treatment of OC, we established a patient-derived xenograft (PDX) model from a patient with chemoresistant (carboplatin- and Taxol-resistant) OC to study the efficacy of DZNep and EPZ6438 **(Supplementary Figure S3B and C)**. The in vivo study indicated that DZNep but not EPZ-6438 significantly suppressed tumor growth **(**Fig. 6D **and E)**. Treatment with either of these inhibitors resulted in a minimal body weight loss, suggesting that both inhibitors had low toxicity **(**Fig. 6F**)**. In DZNep treated tumors, the Ki67 level was concomitantly decreased with that of EZH2 but not with that of H3K27me3, further indicating that the oncogenic role of EZH2 is independent of its catalytic activity **(**Fig. 6G **and Supplementary Figure S3D)**. To further confirm that EZH2 inhibition can suppress the metabolic rewiring, we next examined the α-KG level in PDX tumors upon treatment with EZH2 inhibitors. The data showed that the α-KG level was significantly decreased upon DZNep treatment but not EPZ6438 treatment **(**Fig. 6H**)**. The α-KG level is consistent with the levels of EZH2 and IDH2 **(**Fig. 6G **and Supplementary Figure S3D)**, suggesting that α-KG is a key metabolite in OC that could predict poor prognosis. Collectively, these results demonstrated that inhibitors resulting in a reduction in EZH2 expression but not inhibition of its catalytic activity exhibit a potential therapeutic efficacy in patients with chemoresistant OC.

## Discussion

**Fig. 7:**
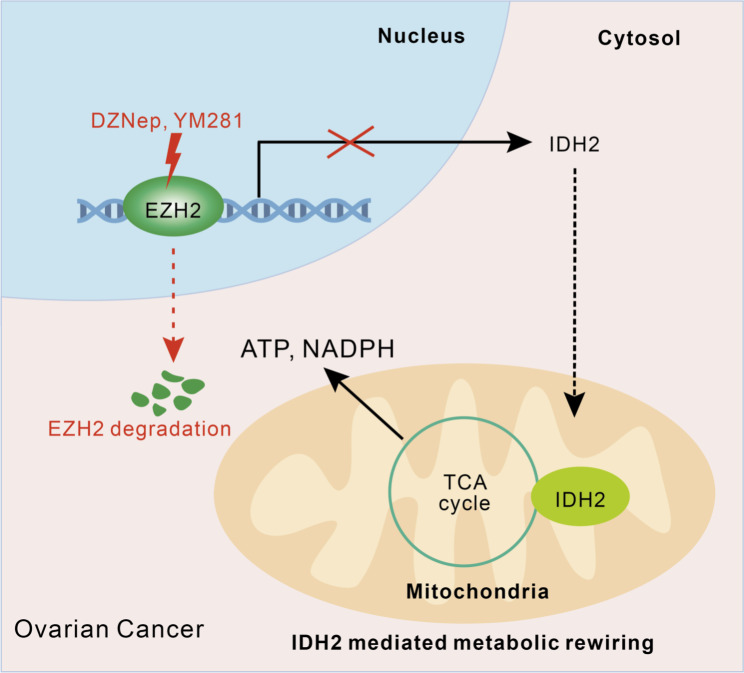
Schematic model illustrating the mechanism of EZH2 potentiating metabolic rewiring in OC Overexpressed EZH2 transcriptionally regulates TCA related genes such as *IDH2*, to enhance the TCA cycle, which promotes metabolic rewiring in OC cells. Small molecules trigger EZH2 degradation, rather than catalytic inhibition, to suppress the transcription of TCA-related genes and significantly block OC growth in vitro and in vivo

EZH2 is gaining attention as an attractive therapeutic target for ovarian cancer [27, 28]. EZH2 is thought to drive tumorigenesis and progression in ovarian cancer through repression of tumor suppressor genes by promoting trimethylation of histone H3K27 (H3K27me3) in the promoter regions, with the expression level of EZH2 being positively correlated with the H3K27me3 level, as *Sun et al.* reported [11]. Other reports have described a high level of H3K27me3 as an indicator of good prognosis; however, H3K27me3 is usually lost in ovarian cancer, contradictory to the oncogenic role of EZH2 as the H3K27me3 methyltransferase [12]. In this study, we showed that a subset of patients with OC with high expression of EZH2 but a low H3K27me3 level exhibited the worst prognosis, which is consistent with previous studies [12, 28]. Since the level of H3K27me3 is hypothesized to be determined by the balance between the activities of the PRC2 complex and the demethylases JMJD3 and UTX, the roles by which JMJD3 and UTX contribute to the level of H3K27me3 in some OC patients warrant further investigation. In other cancers, EZH2 has been reported to activate oncogenic pathways in a PRC2-independent manner [15–17]. In our study, we demonstrated that in OC, EZH2 transcriptionally upregulated *IDH2* to potentiate metabolic rewiring by enhancing tricarboxylic acid cycle (TCA cycle) activity, which provide a novel mechanism by EZH2 to promote tumor growth. Our study improved the understanding of the relationship between EZH2 and H3K27me3 in OC, and began to delve into the mechanisms of the noncanonical roles of EZH2 as a transcriptional activator and its impact on the selection of therapeutic strategies (Fig. 7).

Currently, EZH2 inhibitors are being tested in a number of clinical trials and have been shown to benefit patients with certain hematological malignancies harboring activating mutations in EZH2 [28, 29]. EZH2 inhibitors can be classified into three types based on their mechanisms: inhibitors of EZH2 methyltransferase activity, inhibitors that dissociate the PRC2 complex, and inhibitors triggering EZH2 degradation [20]. The anticancer efficacy of the different classes of EZH2 inhibitors is dependent on the roles of EZH2 in the tumor. In our study, DZNep and YM281, two inhibitors triggering EZH2 degradation, exhibited potent anticancer efficacy in OC cell lines and xenografts, but GSK126 and EPZ-6438, which inhibit the methyltransferase activity of EZH2, did not. Therefore, our findings revealed a noncanonical role of EZH2 in reprogramming metabolism in OC. We further demonstrated in in vivo models that inhibitors triggering EZH2 degradation have promising application in OC treatment, suggesting that the design of more specific, more efficient and safer EZH2 degraders is an important direction in the development of drugs for OC.

The great need for energy to support tumor cell expansion is satisfied by metabolic reprogramming, which is defined as a key hallmark of cancer [30]. Accumulating evidence has revealed that aerobic glycolysis (the typical Warburg effect) is not the sole mechanism that tumor cells preferentially employ to produce energy [31–33]. OXPHOS is also used to produce the amount of energy required for proliferation in some tumor types, such as OC [25]. In our study, we revealed a noncanonical mechanism that implicates EZH2 in the regulation of glucose metabolism. We demonstrated that EZH2 directly promotes *IDH2* transcription to enhance glucose metabolism via OXPHOS in OC cells. IDH2 is an important enzyme in the TCA cycle that catalyzes the production of α-KG. Certain active site mutations in IDH2 enable the production of 2-hydroxyglutarate (2–HG) in place of α-KG, and are frequently found in multiple types of tumors [34]. 2-HG, a cancer cell-autonomous oncometabolite, promotes tumor initiation by modifying DNA and histone methylation [35]. However, such mutations in *IDH2* seldom occur in ovarian cancer, implying that overexpression of *IDH2* promotes OC progression by facilitating TCA cycle activity to produce enough energy and not by producing 2-HG [36]. Moreover, *EZH2* and *IDH2* are overexpressed in ovarian cancer stem cells, which favor OXPHOS over glycolysis for energy production [10, 37]. Interestingly, the TCA cycle intermediate α-KG, the product of IDH2, enables the maintenance of H3K27 hypomethylation [38, 39]. Our study demonstrated that EZH2 transcriptionally upregulates IDH2, which increases the cellular level of α-KG. Therefore, the worse prognosis of patients with the EZH2^high^/H3K27me3^low^ subtype of OC may be due to the collective actions of two pathways: EZH2-driven metabolic rewiring and α-KG-mediated suppression of H3K27me3.

Herein, we observed that EZH2 regulates OXPHOS by promoting IDH2 expression through a methyltransferase independent mechanism, a finding that highlights the importance of noncanonical EZH2 signaling in ovarian carcinogenesis. However, there are several limitations to this study; for example, the noncanonical role of EZH2 discovered herein is found in only approximately one-quarter of OC patients. Furthermore, the mechanism of H3K27 trimethylation in the H3K27me3^high^/EZH2^low^ subgroup remains to be elucidated. Despite these limitations, our study suggests that therapeutic targeting of the EZH2-IDH2 axis might be a promising strategy for combating ovarian cancer. We found several EZH2 inhibitors effective for this purpose, but further clinical validation is needed before their application in OC treatment.

## Conclusion

We demonstrated that EZH2 transcriptionally upregulated IDH2 to potentiate metabolic rewiring by enhancing TCA cycle activity, which contributed to the growth of OC. Our study revealed a noncanonical role of EZH2 in OC and demonstrated that targeting the catalysis-independent activity of EZH2 is a potential therapeutic strategy.

## Electronic supplementary material

Below is the link to the electronic supplementary material.

Supplementary Material 1

## Data Availability

The datasets generated and/or analysed during the current study are available in the Research Data Deposit public platform (www.researchdata.org.cn) with approval number RDDB2023743679.

## Abbreviations

α-KG: α-ketoglutarate
AR: Androgen receptor
ChIP-Seq: Chromatin immunoprecipitation sequencing
EZH2: Enhancer of zeste homolog 2
EZH2-∆SET: EZH2 with SET domain deletion
GSEA: Gene set enrichment analysis
GTEx: Genotype-Tissue Expression
IDH2: Isocitrate dehydrogenase 2
IHC: Immunohistochemistry
NKTL: Natural killer/T-cell lymphoma
OC: Ovarian cancer
OCR: Oxygen consumption rate
OD: Optical density
OXPHOS: Oxidative phosphorylation
PDX: Patient derived xenograft
PRC2: Polycomb repressive complex 2
qRT-PCR: Quantitative real-time PCR
RNA-Seq: RNA sequencing
SAM: S-adenosylmethionine
TCA: Tricarboxylic acid
TCGA: The Tumor Genome Atlas.
