# Supplementary material for: EZH2 mediated metabolic rewiring promotes tumor growth independently of histone methyltransferase activity in ovarian cancer
Source: Mol Cancer. 2023 May 20;22:85. doi: 10.1186/s12943-023-01786-y (PMC10199584; doi:10.1186/s12943-023-01786-y)
Supplement: Supplementary file 1 — Supplementary Material 1 [file 12943_2023_1786_MOESM1_ESM.docx]

**Supplementary Materials**

**Supplemental Methods**

**Clinical samples, cell lines, and reagents**

Informed consent was obtained from the patients who provided the tissue samples, and all procedures were approved by the medical ethics committee of the Sun Yat-sen University Cancer Center (Guangzhou, China). All commercial cell lines were purchased from ATCC, except for COV504 (obtained from ECACC). Authentication of cell lines was performed by the authors. The cell lines were maintained in RPMI 1640 medium (HyClone) supplemented with 10% fetal bovine serum (HyClone) and 1% penicillin/streptomycin (Gibco). Mycoplasma testing was performed using the MycoSensor PCR assay kit (Stratagene). DZNep (S7120), GSK126 (S7061) and EPZ-6438 (S7128) were purchased from Selleck Chemicals. YM281 was a kind gift from Dr. S. Wen (Sun Yat-sen University Cancer Center, Guangzhou, China).

**Establishment of Patient-Derived OC Cells from Primary Tumors**

Tumor cells were disassociated from OC patient-derived primary tumors by collagenase and then seeded and maintained in DMEM/F12 (1:1) medium supplemented with 10% FBS and 1% penicillin/streptomycin. At 80% to 90% confluence, the cells were passaged at a 1:3 ratio.

**IHC staining**

Tumor tissues derived from patients or animal models were fixed with 4% formalin, embedded in paraffin and sectioned. The paraffin sections were incubated with 3% hydrogen peroxide to block endogenous peroxidase activity for 15 minutes at 37 °C and rinsed with 0.01 M PBS prior to high-pressure antigen retrieval in EDTA buffer. The sections were then incubated with antibodies specific for EZH2 (Cell Signaling Technology, 5246), H3K27me3 (Cell Signaling Technology, 9733), IDH2 (Cell Signaling Technology, 60322), and Ki-67 (ZSBiO Commerce Store, ZA-0502) at 4 °C overnight, respectively. After rinsing 3 times in PBS, the sections were incubated with components of a rabbit or mouse polymer detection system kit (ZSBiO Commerce Store, PV-6001/ PV-6002) at room temperature for 1 hour. To determine the specificity of the immunostaining, IgG was used as a negative control. Then, immunoreactivity was detected using a DAB reagent kit (ZSBiO Commerce Store, ZLI-917) according to the manufacturer’s instructions. The immunostaining scores for EZH2, H3K27me3, IDH2 and Ki67 were assessed by microscopy by two pathologists according to the following equation: score = 3 (strong positive) × percentage + 2 (moderate positive) × percentage + 1 (weak positive) × percentage + 0 (negative) × percentage. The percentage value was defined using the following criteria: 0 (< 10% positive cells), 1 (10–25% positive cells), 2 (25–50% positive cells), 3 (50–75% positive cells) and 4 (75–100% positive cells). The cutoff values for high or low EZH2/H3K27me3/IDH2 expression were calculated by using the X-Tile software, which accounts for the presence of substantial tumor subpopulations and shows the robustness of the relationship between a biomarker and an outcome by construction of a two dimensional projection of every possible subpopulation[1]. The EZH2 and H3K27me3 expression pattern subgroups were defined based on the cutoff values.

**Immunoblotting**

Cell lysates were prepared, and proteins in the lysates were separated by SDS-PAGE and transferred to polyvinylidene difluoride membranes. The membranes were blocked in 5% nonfat milk or 5% BSA in Tris-buffered saline (TBS; 10 mmol/L Tris, 10 mmol/L NaCl) and incubated overnight with primary antibodies specific for the following proteins: GAPDH (Cell Signaling Technology, 5174), EZH2 (Cell Signaling Technology, 5246), H3K27me3 (Cell Signaling Technology, 9733), Histone H3 (Cell Signaling Technology, 14269), and IDH2 (Cell Signaling Technology, 60322). Horseradish peroxidase–conjugated anti-rabbit and anti-mouse secondary antibodies (GE Healthcare Life Science, NA934 and NA931) were used. Immunoreactions were detected with ECL Western Blotting Detection Reagents (GE Healthcare Life Science) and a Bio-Rad ChemiDoc MP imaging system. GAPDH and Histone H3 were used as the internal controls for cytosolic and nuclear proteins, respectively.

**Cell proliferation, colony formation, and tumorsphere formation assays**

For the proliferation assay, 2 × 10^3^ cells per well were seeded in a 96-well plate. Twenty-four hours later, the cells were treated with the indicated compounds. The viability of the cells was measured by CellTiter Glo reagent (Promega, G7570) daily. For the colony formation assay, 1 × 10^4^ cells per well were plated in a 6-well plate and incubated with the indicated compounds for 12 days until the cells in the control group were 100% confluent. Colonies were stained with crystal violet (Sigma) for visualization. For the tumorsphere formation assay, a single-cell suspension was plated (1 × 10^4^ cells/well) in 6-well ultralow-attachment plates (Corning) with MammoCult Medium (STEMCELL Technologies) supplemented with heparin (1:500) and fresh hydrocortisone (0.5 μg/mL). Tumorspheres were cultured for 10-12 days and photographed. Imaging and quantification were performed using Olympus CellSens Dimension software and the connected Olympus microscope IX71.

**Plasmid construction, virus production and infection**

The Tet-pLKO-puro cloning vector (Addgene, Plasmid #21915) was employed to construct the scramble (SCR) and *EZH2* shRNA plasmids. The proteins encoded by the human *EZH2* and *EZH2-ΔSET* sequences were expressed via the pBMN-I-GFP retroviral expression vector (gifts from Q. Yu at Genome Institute of Singapore, Agency for Science, Technology and Research (A*STAR), Biopolis, Singapore) as described previously[2]. A silent point mutation was generated at the *EZH2*#2 shRNA target site in the cloned *EZH2* and *EZH2-ΔSET* gene sequences using the KOD-Plus-Mutagenesis Kit (TOYOBO, SMK-101). The coding sequence (CDS) of human *IDH2* was amplified by PCR from pooled cDNA of OVCAR8 cells and was inserted into the pCDH cDNA cloning and expression lentivector (System Biosciences, cat# CD526A-1) via the EcoRI and BamHI endonuclease sites. The *IDH2* sequence from -1000bp upstream of the TSS through the 5’UTR was amplified and inserted into the pGL3-basic plasmid via the KpnI and XhoI endonuclease sites. The lentiviral vectors were transfected into 293T cells with the packaging vectors psPAX2 and pMD2.G. Then, the resulting lentiviral particles were harvested for infection of the target cells 48 hours later. The retroviral vectors were transfected into PlatA packaging cells. Forty-eight hours post-transfection, viral supernatants were passed through a 0.45 μm nitrocellulose filter and were used to infect OC cells in the presence of polybrene (8 μg/ml). Stable retroviral cell lines were selected by sorting on GFP for further analysis. The primer sequences used for plasmid construction are shown in Supplementary **Table S1**.

**RNA sequencing and analysis**

RNA was collected from OVCAR8 and OVCAR8-sh*EZH2*#2 cells treated with DMSO, DZNep (5 μM), GSK126 (5 μM) or doxycycline (1 μg/mL) for 72 hours. Total RNA was extracted using TRIzol Reagent (Invitrogen) and purified with an RNeasy Mini Kit (QIAGEN). Transcriptome sequencing libraries were prepared using a TruSeq Stranded RNA HT kit (Illumina, 15032620) according to the manufacturer’s protocol. Briefly, ribosomal RNA was removed by beads, and the remaining RNA was fragmented into small pieces and reverse transcribed to single-stranded cDNA. The cDNA was ligated to adaptors and was then amplified by the standard Illumina library preparation method. Samples were sequenced on the Illumina NovaSeq6000 platform according to the manufacturer’s protocol. For data analysis, fastp software (version 0.12.5) was used to determine the quality of the original data, and the adaptors were removed[3]. All the clean reads were aligned to the human reference genome (GRCh38, hg38) with STAR with GENCODE annotations (gencode.v37.annotation.gtf). RSEM was used to quantify gene expression, and edgeR[4] (log2FoldChange > 1, *P* < 0.05) was used to identify differentially expressed genes. All further analyses were performed using R statistical software. The RNA-Seq data reported in this study are accessible in the NCBI GEO database under GEO Series accession number GSE185604.

**Quantitative real-time PCR (qRT-PCR)**

Total RNA was extracted by using TRIzol and purified with an RNAeasy Mini Kit (QIAGEN). Reverse transcription and quantitative PCR assays were performed using the TransScript All-in-One First-Strand cDNA Synthesis SuperMix for qPCR kit (Transgene Biotech) and KAPA SYBR FAST qPCR Master Mix (2×) kit (Sigma-Aldrich). For the quantification of mRNA expression, the 18S rRNA level was used as an internal control. Reactions were performed in a Real-Time PCR Detection System (Bio-Rad). All experiments were performed in biological triplicates unless stated otherwise. The primer sequences used for qRT-PCR are shown in **Supplementary** **Table S1**.

**Dual luciferase reporter assay**

The day prior to transfection, 1×10^4^ cells were seeded into each well of 96 well plates. The cells were transfected with 100 ng of pGL3-*IDH2*-promoter and 5 ng of pRL-TK (Renilla luciferase vector, Promega) per well. The cells were treated with DZNep (1 μM) and GSK126 (5 μM) separately 6 hours after transfection. The cells were lysed and analyzed using the Dual-Luciferase Reporter System (Promega) 48 hours later.

**ChIP and ChIP-qPCR**

OVCAR8 cells were subjected to crosslinking with 1% formaldehyde for 10 minutes at room temperature, and the reaction was quenched by incubation with 0.125 M glycine for 5 minutes. After crosslinking, the cells were washed with TBSE buffer (20 mM Tris-HCl pH 7.5, 1 mM EDTA, 150 mM NaCl). The cells were then lysed (50 mM Tris-HCl, pH 8.0; 10 mM EDTA; 1% SDS) and sonicated on ice using a Bioruptor sonicator (Diagenode, 50 cycles for EZH2 and SUZ12, 16 cycles for H3K27me3 and H3K4me3, 30 seconds on and 30 seconds off). The sonicated lysates were precleared with Protein G Dynabeads (Invitrogen) for 1 hour at 4 °C and were incubated overnight at 4 °C with magnetic beads conjugated to antibodies against EZH2 (Diagenode, MB-180-050), H3K27me3 (Cell Signaling Technology, 9733), H3K4me3 (Cell Signaling Technology, 9751) and SUZ12 (Abcam, ab12073) and with normal rabbit IgG (EMD Millipore 12-370). The immunoprecipitates were washed 4 times, and proteins were eluted in elution buffer (50 mM Tris-HCl, pH 8.0; 10 mM EDTA; 1% SDS) for subsequent reversal of cross-linking by incubation with pronase at 42 °C for 2 hours and 68 °C for 6 hours. The uncrosslinked DNA was purified by phenol–chloroform–isoamyl alcohol (Thermo Fisher Scientific) and ethanol precipitation. ChIP-qPCR was performed using SYBR Green PCR Master Mix (Applied Biosystems). The primer sequences are listed in **Supplementary Table S1**. The enrichment of specific genomic regions was calculated relative to the input DNA, followed by normalization to the values in the respective control IgG groups.

**ChIP-Seq and ChIP-Seq data analysis**

Whole-genome amplification was performed using the WGA4 kit (Sigma-Aldrich) and BpmI-WGA primers. Amplified DNA was digested with BpmI (New England Biolabs). ChIP DNA (30 ng) was used for library construction using the NEBNext ChIP-Seq Library Prep Master Mix Set for Illumina (New England Biolabs) according to the manufacturer’s recommended protocol. Libraries were sequenced using an Illumina HiSeq 2500 system. ChIP-Seq data analysis was performed as previously described[5]. In brief, the quality of the raw data was verified using FastQC (v0.11.9; default parameters) and adapter sequences were removed using Trim Galore (version 0.6.6, https://www.bioinformatics.babraham.ac.uk/projects/trim_galore/)[6]. Reads were mapped to the human genome hg19 (hs37d5) using Bowtie2 (version 2.3.5.1)[7]. Reads with a mapping quality higher than 10 were retained and sorted using SAMtools (version 1.9), after which duplicates were removed using the Picard toolkit (MarkDuplicates version 2.20.3, http://broadinstitute.github.io/picard/)[8]. MACS2 was used for peak identification with data from the input samples used as the control and with the default parameters[9]. Alignment files in bam format were converted into read coverage files (bigwig format) using DeepTools (v3.5.1)[10]. Profiles of ChIP-seq read densities were visualized in Integrative Genomics Viewer (IGV, Broad Institute). Peaks were annotated with ChIPseeker (version 1.20.0)[11]. EZH2 solo peaks, EZH2 ensemble peaks and H3K27me3 solo peaks were identified using the BEDTools (v2.30.0) interset function[12]. Heatmaps of ChIP-seq signals were generated using the DeepTools computeMatrix and plotHeatmap functions. The ChIP-Seq data reported in this study are accessible in the NCBI GEO database under the GEO Series accession number GSE211307.

**Oxygen consumption rate measurement**

The cellular oxygen consumption rate (OCR) was determined using the Seahorse XFe 96 Extracellular Flux Analyzer (Seahorse Bioscience). Experiments were performed according to the manufacturer's protocols. The OCR was measured using the Seahorse XF Cell Mito Stress Test Kit (Agilent Technologies). Briefly, 2 × 10^4^ cells per well were seeded into a Seahorse XF 96 cell culture microplate. Oligomycin, the reversible inhibitor of oxidative phosphorylation FCCP (*p*‐trifluoromethoxy carbonyl cyanide phenylhydrazone), and the mitochondrial complex I inhibitor rotenone, plus the mitochondrial complex III inhibitor antimycin A (Rote/AA), were injected sequentially to measure basal OCR, ATP content, maximal OCR, and non-mitochondrial OCR. Data were analyzed using Seahorse XF‐96 Wave (Agilent Technologies) software. The OCR values are shown in pmols/min.

**α-Ketoglutarate assay**

The tissue and cellular α-ketoglutarate (α-KG) levels were determined using the α-ketoglutarate colorimetric/fluorometric assay kit (BioVision). Experiments were performed according to the manufacturer's protocols. Briefly, 2× 10^6^ cells per sample were harvested and rapidly homogenized with 100 µl of ice cold α-KG assay buffer. Samples were deproteinized using 10 kDa molecular weight cut off spin columns (BioVision). After 10 µl samples added into duplicate wells of a 96-well plate, assay buffer was used to bring the volume to 50 µl. Enough reagent for the number of samples and standards was mixed and incubated for 30 min at 37 °C in the dark. The optical density (OD) values were measured at 570 nm. α-KG concentrations are shown in nmol/µL.

***In vivo* studies**

All animal studies were conducted in compliance with animal protocols approved by the Institutional Animal Care and Use Committee of Sun Yat-sen University Cancer Center (Guangzhou, China). Female BALB/c nude mice (5–6 weeks old) were purchased from Beijing Vital River Laboratory Animal Technology Company. Tumors were measured with Vernier calipers, and the tumor volume was calculated with the following formula: tumor volume = length × width^2^/2. When the tumor volume reached approximately 100 mm^3^, the mice were divided into different groups for treatment. Randomization was performed by equally dividing the tumor-bearing mice with a similar tumor burden into groups for drug treatment. For the EZH2-inducible knockdown xenograft assay, mice were implanted subcutaneously in the flank with 2 × 10^6^ OVCAR8 cells with inducible sh-scramble (shSCR) or sh*EZH2* expression. Doxycycline was dissolved in distilled water and administered by oral gavage at 150 mg/kg daily. For the treatment with DZNep and EPZ-6438, mice were implanted subcutaneously in the right flank with 5 × 10^6^ OVCAR8 cells. For the PDX models, PDX-POVC15 tumor masses were passaged in NOD/SCID mice after subcutaneous implantation. When the tumor volumes reached approximately 100 mm^3^, the mice were randomly divided into three groups. DZNep was suspended in 1× saline and was given twice a week by intraperitoneal injection (1 mg/kg). EPZ-6438 was dissolved in vehicle consisting of 0.5% weight/volume sodium carboxymethylcellulose and 0.1% volume/volume Tween 80 and given by oral gavage (40 mg/kg daily). For the treatment with YM281, mice were administered vehicle control (80% PBS, 10% castor oil, and 10% DMSO) or YM281 (80 mg/kg) through intraperitoneal injection 6 times weekly. Tumor volume and body weight were monitored twice a week until the tumor volume reached 1,000 mm^3^. Mice were sacrificed by CO_2_ inhalation, and tumors were harvested for further analysis.

**Statistical analysis**

The data are presented as the mean ± SD values unless otherwise specified. Significant differences between two groups were identified using two-tailed Student’s t test for unpaired data, and *P*<0.05 was considered to indicate a statistically significant difference. All statistical analyses were performed by GraphPad Prism 7 (La Jolla, CA).

**References：**

1. Camp RL, Dolled-Filhart M, Rimm DL: **X-tile: a new bio-informatics tool for biomarker assessment and outcome-based cut-point optimization.** *Clin Cancer Res* 2004, **10:**7252-7259.

2. Yan J, Ng SB, Tay JL, Lin B, Koh TL, Tan J, Selvarajan V, Liu SC, Bi C, Wang S, et al: **EZH2 overexpression in natural killer/T-cell lymphoma confers growth advantage independently of histone methyltransferase activity.** *Blood* 2013, **121:**4512-4520.

3. Chen S, Zhou Y, Chen Y, Gu J: **fastp: an ultra-fast all-in-one FASTQ preprocessor.** *Bioinformatics* 2018, **34:**i884-i890.

4. McCarthy DJ, Chen Y, Smyth GK: **Differential expression analysis of multifactor RNA-Seq experiments with respect to biological variation.** *Nucleic Acids Res* 2012, **40:**4288-4297.

5. Liu S, Zou Q, Chen JP, Yao X, Guan P, Liang W, Deng P, Lai X, Yin J, Chen J, et al: **Targeting enhancer reprogramming to mitigate MEK inhibitor resistance in preclinical models of advanced ovarian cancer.** *J Clin Invest* 2021, **131**.

6. Ward CM, To TH, Pederson SM: **ngsReports: a Bioconductor package for managing FastQC reports and other NGS related log files.** *Bioinformatics* 2020, **36:**2587-2588.

7. Langmead B, Salzberg SL: **Fast gapped-read alignment with Bowtie 2.** *Nat Methods* 2012, **9:**357-359.

8. Danecek P, Bonfield JK, Liddle J, Marshall J, Ohan V, Pollard MO, Whitwham A, Keane T, McCarthy SA, Davies RM, Li H: **Twelve years of SAMtools and BCFtools.** *Gigascience* 2021, **10**.

9. Zhang Y, Liu T, Meyer CA, Eeckhoute J, Johnson DS, Bernstein BE, Nusbaum C, Myers RM, Brown M, Li W, Liu XS: **Model-based analysis of ChIP-Seq (MACS).** *Genome Biol* 2008, **9:**R137.

10. Ramirez F, Ryan DP, Gruning B, Bhardwaj V, Kilpert F, Richter AS, Heyne S, Dundar F, Manke T: **deepTools2: a next generation web server for deep-sequencing data analysis.** *Nucleic Acids Res* 2016, **44:**W160-165.

11. Yu G, Wang LG, He QY: **ChIPseeker: an R/Bioconductor package for ChIP peak annotation, comparison and visualization.** *Bioinformatics* 2015, **31:**2382-2383.

12. Quinlan AR, Hall IM: **BEDTools: a flexible suite of utilities for comparing genomic features.** *Bioinformatics* 2010, **26:**841-842.

**Supplementary Figures and Legends**

**
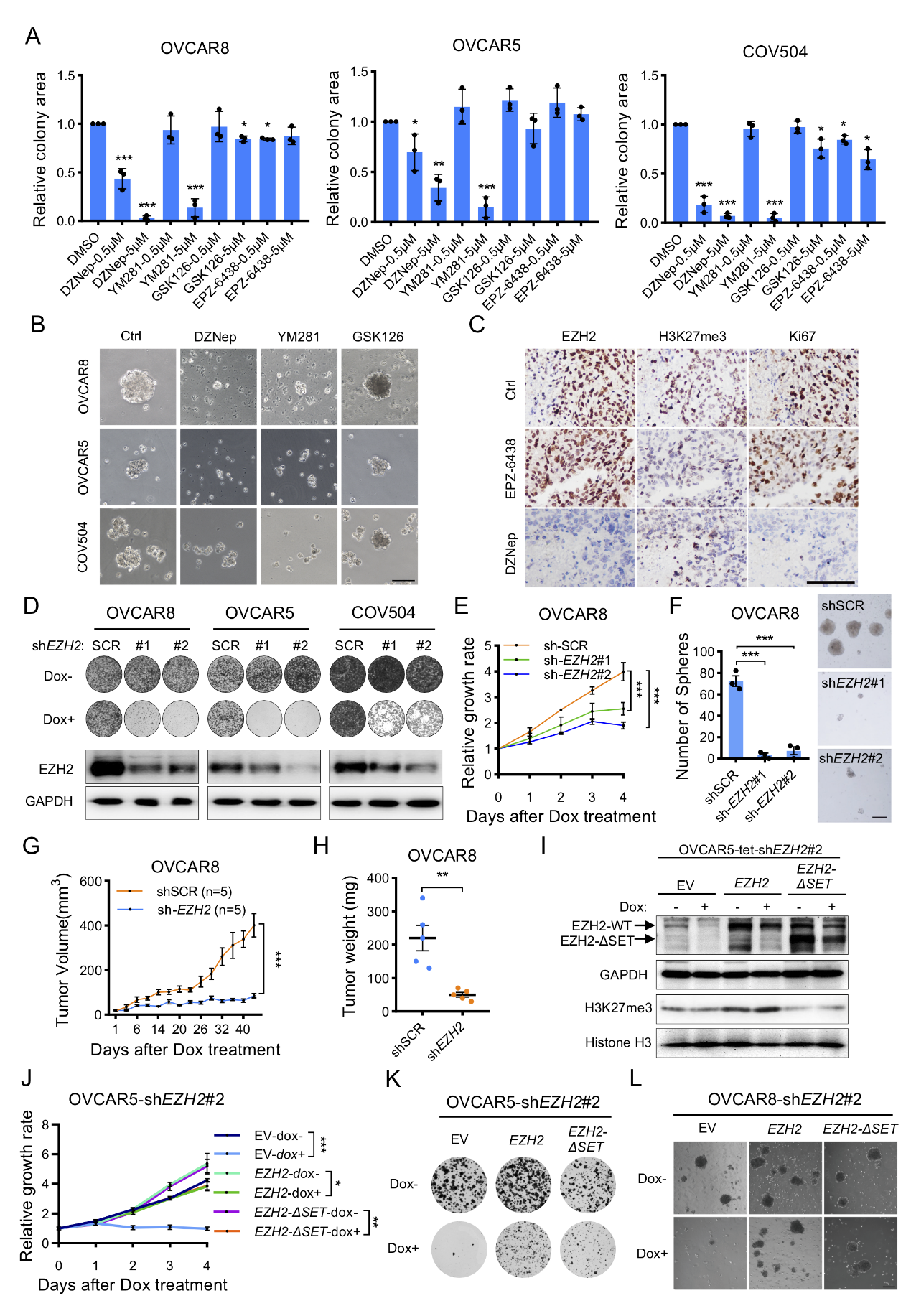
**

**
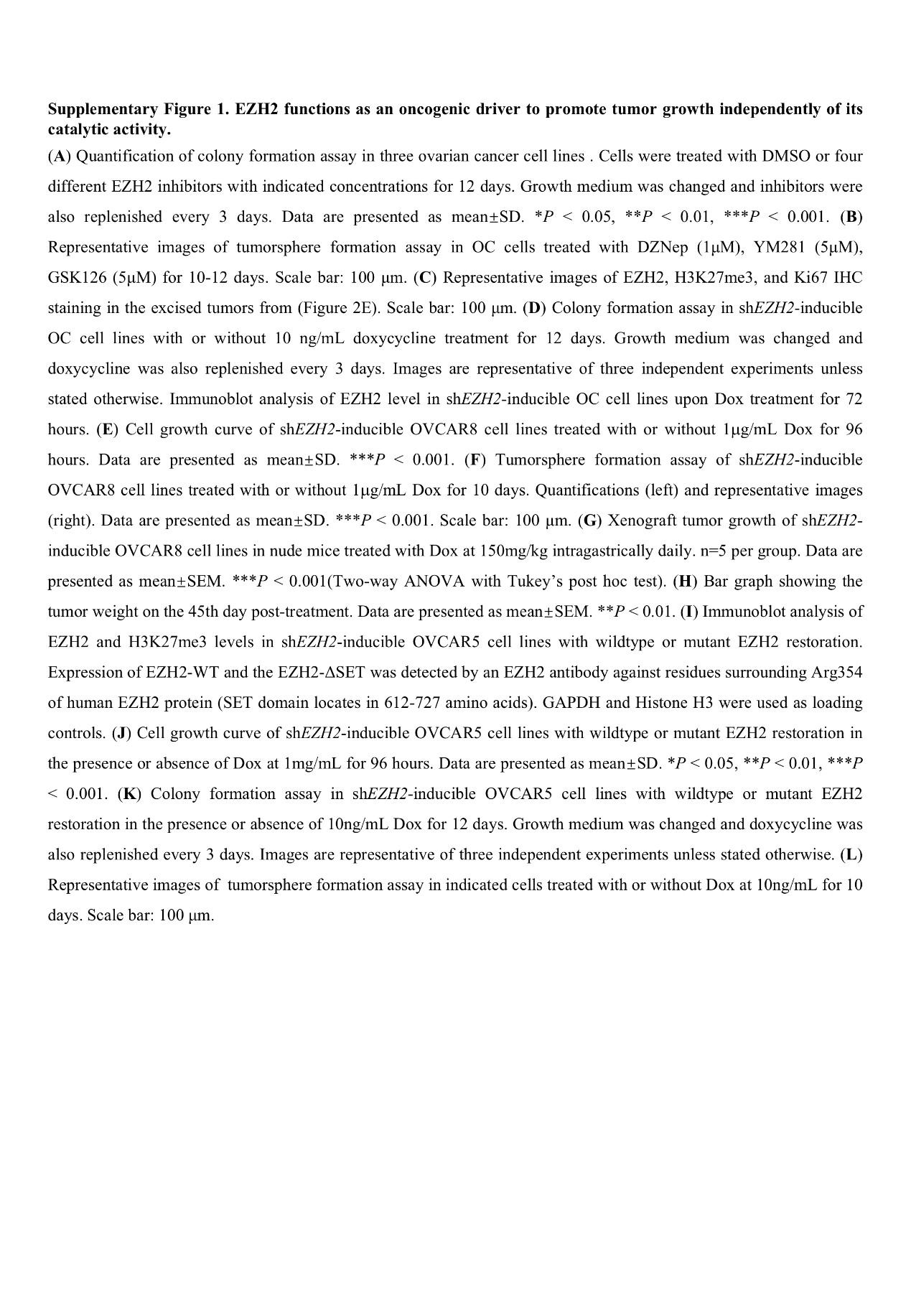

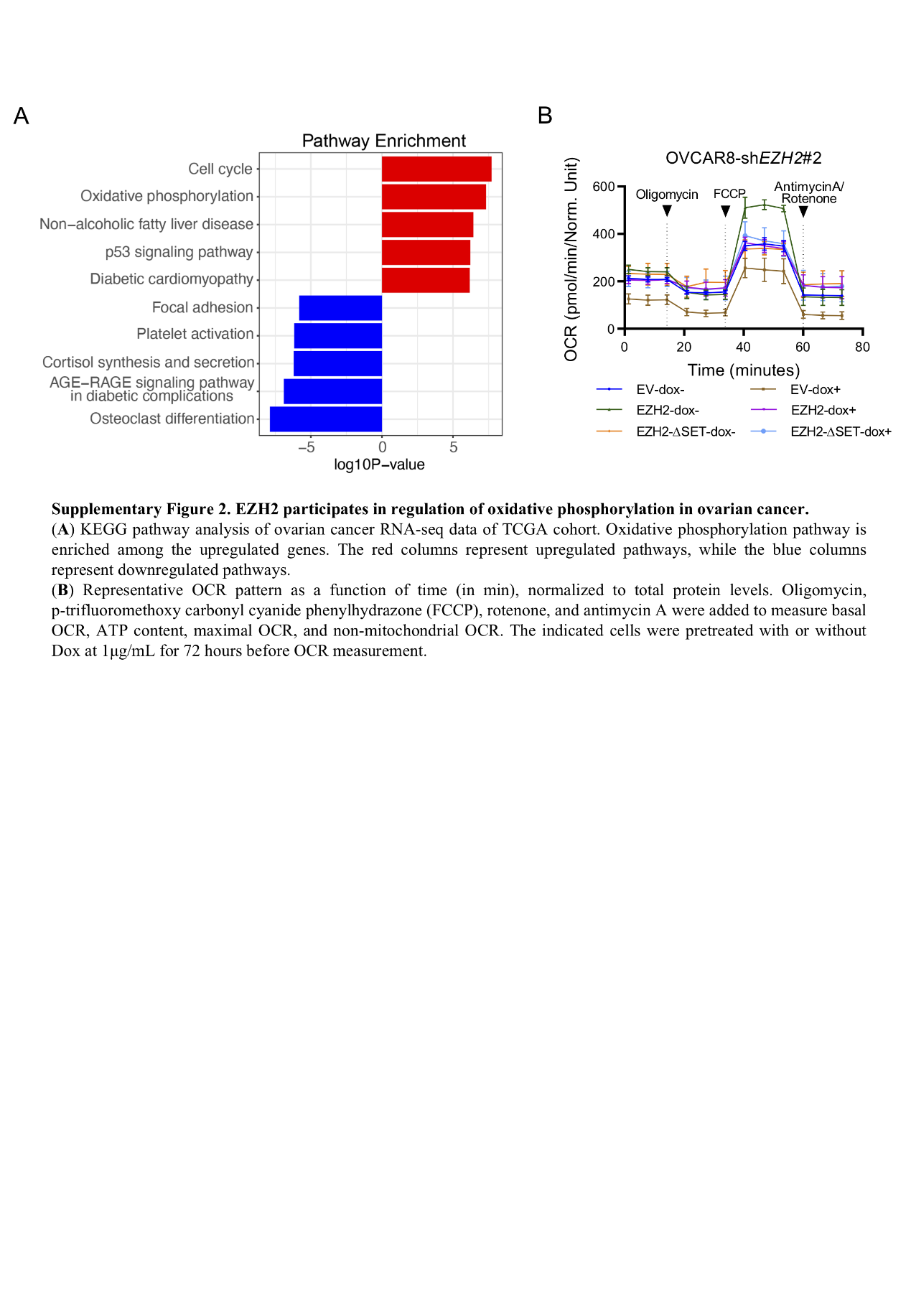

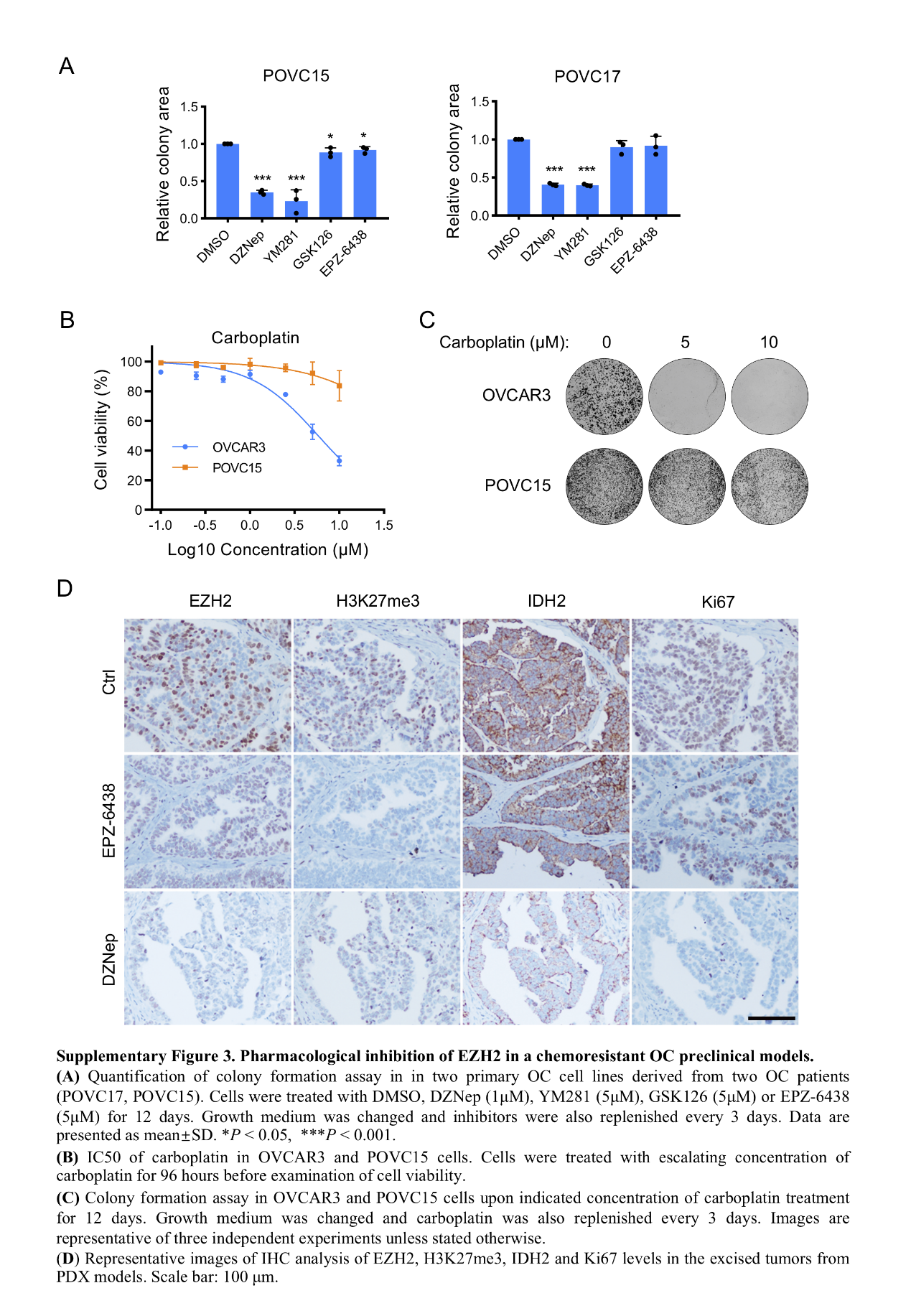
**

**Supplementary Table**

**Table S1. The sequences of primers, shRNA and siRNA used in this study**

| **Primers** | **5' to 3'** |
| --- | --- |
| *EZH2* qRT-PCR forward primer | CCTCATTGGCACTTACTA |
| *EZH2* qRT-PCR reverse primer | AACTCATACACCTGTCTAC |
| *IDH2* qRT-PCR forward primer | CGCCACTATGCCGACAAAAG |
| *IDH2* qRT-PCR reverse primer | ACTGCCAGATAATACGGGTCA |
| *OGDHL* qRT-PCR forward primer | CATCGACAAATCCAGCGAGAT |
| *OGDHL* qRT-PCR reverse primer | ATCCTCTCATGGTACATGCCC |
| *PCK2* qRT-PCR forward primer | GCCATCATGCCGTAGCATC |
| *PCK2* qRT-PCR reverse primer | AGCCTCAGTTCCATCACAGAT |
| *IDH2* P1 ChIP-qPCR forward primer | CCTGCGGAGCTGGGCTTT |
| *IDH2* P1 ChIP-qPCR reverse primer | AACGCTGGGCCTGGCGGG |
| *IDH2* P2 ChIP-qPCR forward primer | CTAATGCGCCTTTGGGTTCAG |
| *IDH2* P2 ChIP-qPCR reverse primer | TACAGGCGTGAGACCCACC |
| *IDH2* P3 ChIP-qPCR forward primer | AGCCTGGTCTCGAACTCCT |
| *IDH2* P3 ChIP-qPCR reverse primer | CCAGTGCGGCTGCTTCATT |
| *IDH2* CDS forward primer | ATGGCCGGCTACCTGCGG |
| *IDH2* CDS reverse primer | CTACTGCCTGCCCAGGGC |
| *IDH2* promoter+5’UTR forward primer | TCTGACCTACCTTGTTTGCTT |
| *IDH2* promoter+5’UTR reverse primer | AAGCTGGAGAGCGAACGA |
| *EZH2* shRNA#1 forward primer | CCGGAAGACTCTGAATGCAGTTGCTCTCGAGAGCAACTGCATTCAGAGTCTTTTTTTG |
| *EZH2* shRNA#1 reverse primer | AATTCAAAAAAAGACTCTGAATGCAGTTGCTCTCGAGAGCAACTGCATTCAGAGTCTT |
| *EZH2* shRNA#2 forward primer | CCGGCCAACACAAGTCATCCCATTACTCGAGTAATGGGATGACTTGTGTTGGTTTTTG |
| *EZH2* shRNA#2 reverse primer | AATTCAAAAACCAACACAAGTCATCCCATTACTCGAGTAATGGGATGACTTGTGTTGG |
| scramble shRNA forward primer | AATTCCTAAGGTTAAGTCGCCCTCGCTCGAGCGAGGGCGACTTAACCTTAGGTTTTTTT |
| scramble shRNA reverse primer | AAAAAAACCTAAGGTTAAGTCGCCCTCGCTCGAGCGAGGGCGACTTAACCTTAGG |
| si-*EZH2*#1 | GCUAGGUUAAUUGGGACCAAA |
| si-*EZH2*#2 | CCCAACAUAGAUGGACCAAAU |
| si-*IDH2*#1 | GCAAGAACUAUGACGGAGAUU |
| si-*IDH2*#2 | AGGCAGGAGCAGUGCGUUUUU |
